# Glucocorticoids inhibit macrophage differentiation towards a pro-inflammatory phenotype upon wounding without affecting their migration

**DOI:** 10.1242/dmm.037887

**Published:** 2019-05-30

**Authors:** Yufei Xie, Sofie Tolmeijer, Jelle M. Oskam, Tijs Tonkens, Annemarie H. Meijer, Marcel J. M. Schaaf

**Affiliations:** Animal Science and Health Cluster, Institute of Biology, Leiden University, 2333CC Leiden, The Netherlands

**Keywords:** Glucocorticoids, Inflammation, Macrophage differentiation, Leukocyte migration, Zebrafish, Tail amputation

## Abstract

Glucocorticoid drugs are widely used to treat immune-related diseases, but their use is limited by side effects and by resistance, which especially occurs in macrophage-dominated diseases. In order to improve glucocorticoid therapies, more research is required into the mechanisms of glucocorticoid action. In the present study, we have used a zebrafish model for inflammation to study glucocorticoid effects on the innate immune response. In zebrafish larvae, the migration of neutrophils towards a site of injury is inhibited upon glucocorticoid treatment, whereas migration of macrophages is glucocorticoid resistant. We show that wounding-induced increases in the expression of genes that encode neutrophil-specific chemoattractants (Il8 and Cxcl18b) are attenuated by the synthetic glucocorticoid beclomethasone, but that beclomethasone does not attenuate the induction of the genes encoding Ccl2 and Cxcl11aa, which are required for macrophage recruitment. RNA sequencing on FACS-sorted macrophages shows that the vast majority of the wounding-induced transcriptional changes in these cells are inhibited by beclomethasone, whereas only a small subset is glucocorticoid-insensitive. As a result, beclomethasone decreases the number of macrophages that differentiate towards a pro-inflammatory (M1) phenotype, which we demonstrated using a *tnfa:eGFP-F* reporter line and analysis of macrophage morphology. We conclude that differentiation and migration of macrophages are regulated independently, and that glucocorticoids leave the chemotactic migration of macrophages unaffected, but exert their anti-inflammatory effect on these cells by inhibiting their differentiation to an M1 phenotype. The resistance of macrophage-dominated diseases to glucocorticoid therapy can therefore not be attributed to an intrinsic insensitivity of macrophages to glucocorticoids.

## Abstract (precis)

**Summary:** In a zebrafish model for inflammation, glucocorticoids do not affect the migration of macrophages, but inhibit their differentiation towards an M1 phenotype, by strongly attenuating transcriptional responses in these cells.

## INTRODUCTION

Glucocorticoids are a class of steroid hormones secreted by the adrenal gland, and the main endogenous glucocorticoid in our body is cortisol (Chrousos, 1995; Oakley and Cidlowski, 2013; Tsigos and Chrousos, 2002). Glucocorticoids regulate a wide variety of systems in our body, including the immune, metabolic, reproductive, cardiovascular and central nervous systems (Chrousos and Kino, 2005; Heitzer et al., 2007; Ramamoorthy and Cidlowski, 2013; Revollo and Cidlowski, 2009). Owing to their potent and well-established immunosuppressive effects, they are often prescribed to treat various immune-related diseases, including asthma, rheumatoid arthritis, dermatitis, leukemia and several autoimmune diseases (Barnes, 2011; Busillo and Cidlowski, 2013). However, their clinical use is limited by two issues. First, chronic glucocorticoid therapy can lead to severe side effects, such as osteoporosis, muscle weakness, diabetes, infection and hypertension (Moghadam-Kia and Werth, 2010). Second, resistance to glucocorticoid drug treatment occurs in a large number (∼10-30%) of patients (Barnes and Adcock, 2009; Barnes et al., 2004). In order to develop novel glucocorticoid therapies that overcome these barriers and retain their therapeutic efficacy, more insight into the molecular and cellular mechanisms of glucocorticoid modulation of the immune response is required.

Glucocorticoids exert their function through an intracellular receptor, the glucocorticoid receptor (GR) (Bamberger et al., 1996), which acts as a transcription factor, altering the transcription of a plethora of genes. The GR modulates the transcription of genes by several mechanisms (Ratman et al., 2013). It can bind directly to glucocorticoid response elements (GRE) in the DNA, and it can enhance transcription upon recruitment of transcriptional cofactors. In contrast, binding to negative GREs (nGREs) has been shown to repress gene transcription (Surjit et al., 2011). Alternatively, the GR can bind indirectly to DNA through interaction with other transcription factors, such as AP-1, NF-κB or STAT3. Through this ‘tethering’, it modulates the activity of these factors.

The tethering mechanism of the GR, resulting in the inhibition of transcription of immune-activating genes, is generally considered to be the main mechanism by which glucocorticoids exert their anti-inflammatory actions (Reichardt et al., 2001). For example, tumor necrosis factor (TNF)- or lipopolysaccharide (LPS)-induced transcriptional responses in cultured cells can be repressed through tethering of the NF-κB subunit p65 (Kuznetsova et al., 2015; Ogawa et al., 2005; Rao et al., 2011; Sacta et al., 2018). Other mechanisms, such as the activation of anti-inflammatory genes through GRE binding, and a reduction of NF-κB recruitment, contribute to the anti-inflammatory actions of GR as well, but the exact role of these mechanisms has not been fully established (Hübner et al., 2015; Oh et al., 2017). Through these mechanisms, glucocorticoids exert strong suppressive effects on the inflammatory response (Smoak and Cidlowski, 2004). At the initial stage of this response, they dampen signaling pathways downstream from Toll-like receptors (TLRs), inhibit the induction of genes encoding cytokines, upregulate the expression of anti-inflammatory proteins and inhibit the generation of prostaglandins and leukotrienes (Busillo and Cidlowski, 2013; Coutinho and Chapman, 2011). In addition, they reduce the blood flow to the inflamed tissue and inhibit vascular leakage. At subsequent stages, glucocorticoids attenuate the production of chemokines and adhesion molecules, thereby reducing leukocyte extravasation and migration towards the inflamed site (Coutinho and Chapman, 2011; Smoak and Cidlowski, 2004).

It has become clear that glucocorticoid action on the immune system is highly complex and requires further investigation. A complicating factor is that the effects of glucocorticoids have been shown to be highly cell type-specific (Franco et al., 2019). Whereas they induce apoptosis of eosinophils and basophils, they promote the survival and proliferation of neutrophils (Meagher et al., 1996; Yoshimura et al., 2001). In monocytes, they induce an anti-inflammatory phenotype with increased mobility and phagocytic capacity (Ehrchen et al., 2007). Macrophages are often divided into two functional phenotypes: a classically activated, pro-inflammatory (M1) phenotype that contributes to the inflammatory response, and an alternatively activated (M2) phenotype that can be subdivided in several different phenotypes, which have been shown to be involved in the resolution of inflammation and wound healing (Martinez and Gordon, 2014; Mosser and Edwards, 2008). In animal models for arthritis and acute lung injury, glucocorticoids have been shown to inhibit the differentiation of macrophages towards an M1 phenotype, whereas the effect on M2 differentiation is less clear (Hofkens et al., 2013; Tu et al., 2017). In addition to the cell type-specificity of glucocorticoid actions, it has become clear that the transcriptional regulation of immune-activating genes by the GR is not strictly suppressive (Cruz-Topete and Cidlowski, 2015). Upregulation of various pro-inflammatory genes after glucocorticoid treatment has been observed in several cell types (Busillo et al., 2011; Chinenov and Rogatsky, 2007; Ding et al., 2010; Galon et al., 2002; Lannan et al., 2012) and the GR has been shown to activate pro-inflammatory genes in synergy with other signaling pathways (Dittrich et al., 2012; Langlais et al., 2008; Langlais et al., 2012). In addition, some genes that are induced upon TNF or LPS treatment appear to be insensitive to the repressive action of GR (Kuznetsova et al., 2015; Ogawa et al., 2005; Rao et al., 2011; Sacta et al., 2018).

In the present study, we have used the zebrafish as an *in vivo* model to study glucocorticoid effects on the inflammatory response. The immune system of the zebrafish is highly similar to that of humans. As in humans, the zebrafish has a thymus, innate immune cells (macrophages, neutrophils) and adaptive immune cells (T cells and B cells), and cells that bridge innate and adaptive immunity (dendritic cells) (Lewis et al., 2014; Masud et al., 2017; Sullivan et al., 2017). Besides, the innate immune system of the zebrafish develops within a few days after fertilization, whereas the adaptive immune system only matures after two weeks, which means the innate immune system can be studied separately in larvae (Masud et al., 2017; Trede et al., 2004). Zebrafish larvae are widely used as a model system to study the inflammatory response (Enyedi et al., 2016; Oehlers et al., 2017; Powell et al., 2017). Tail wounding-induced inflammation in zebrafish larvae is a well-established model in which amputation of the tail triggers the expression of many pro-inflammatory molecules and the recruitment of innate immune cells (neutrophils and macrophages) towards the wounded area (Renshaw et al., 2006; Roehl, 2018). This model enables the investigation of cell type-specific inflammatory responses *in vivo* and has been widely used for research on leukocyte migration and infiltration, and anti-inflammatory drug screening (Niethammer et al., 2009; Robertson et al., 2016; Yoo et al., 2011).

The zebrafish Gr is highly similar to its human equivalent in structure and function (Chatzopoulou et al., 2015; Schaaf et al., 2008; Stolte et al., 2006). This makes the zebrafish a valuable model to study the molecular mechanisms of glucocorticoid action *in vivo* (Alsop and Vijayan, 2008; Schaaf et al., 2008; Schaaf et al., 2009). In previous work, we have studied the anti-inflammatory effects of glucocorticoids using the tail amputation model and found that glucocorticoid treatment attenuates the vast majority amputation-induced changes in gene expression, which were measured in lysates from whole larvae (Chatzopoulou et al., 2016). In addition, we observed that the recruitment of neutrophils to the wounded area is inhibited by glucocorticoids, but that the migration of macrophages is resistant to glucocorticoid treatment (Chatzopoulou et al., 2016; Mathew et al., 2007; Zhang et al., 2008).

It has been shown that glucocorticoids are less effective in the treatment of inflammatory diseases dominated by macrophages, such as chronic obstructive pulmonary disease (COPD), but the mechanisms underlying the limited responsiveness to glucocorticoid treatment remain poorly understood (Hakim et al., 2012). Therefore, in the present study, we sought to find a mechanistic explanation for our finding that glucocorticoids do not inhibit amputation-induced macrophage migration. We demonstrate that the induction of genes encoding chemoattractants involved in macrophage recruitment is insensitive to glucocorticoid treatment, providing an explanation for the resistance of macrophage migration to glucocorticoids. In addition, we show that macrophages should not be considered a generally glucocorticoid-insensitive cell type. In these cells, glucocorticoids attenuate almost all wounding-induced changes in gene expression. Through this modulation of the transcriptional response, glucocorticoids inhibit the differentiation of macrophages to a pro-inflammatory (M1) phenotype.

## RESULTS

### Glucocorticoids inhibit migration of neutrophils, but leave macrophage migration unaffected

**Fig. 1.**
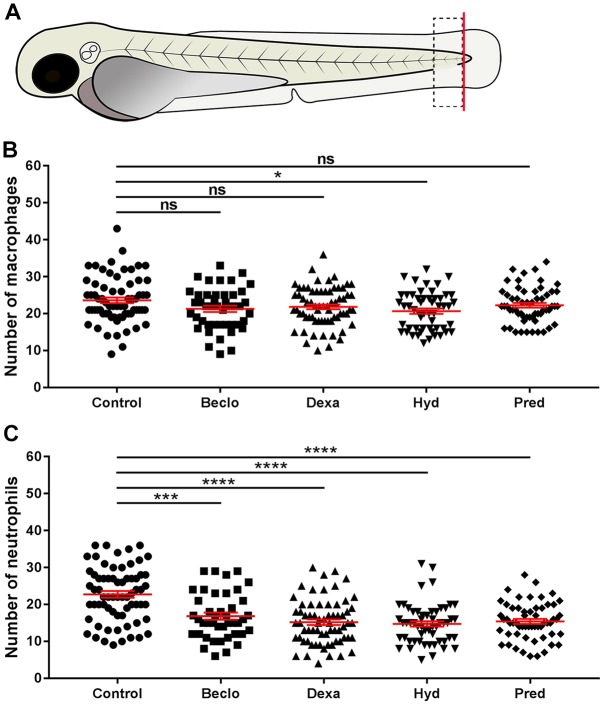
**Effect of glucocorticoids on macrophage and neutrophil recruitment upon tail amputation in *Tg*(*mpx:GFP/mpeg1:mCherry-F*) larvae.** (A) Schematic drawing of a zebrafish larva at 3 dpf. The red line shows the site of amputation. The black dashed box shows the area in which cells were counted to quantitate the recruitment. (B) The number of macrophages recruited to the wounded area at 4 hpa. In the beclomethasone (Beclo), dexamethasone (Dexa) and prednisolone (Pred) groups, no significant differences were observed compared the vehicle-treated (control) group. In the hydrocortisone (Hyd) group, a significantly decreased number of macrophages was observed. (C) The number of neutrophils recruited to the wounded area at 4 hpa. For all glucocorticoid-treated groups, a significantly reduced number of neutrophils was recruited compared to the control group. Data are mean±s.e.m. from three independent experiments. *P<0.05; ***P<0.001; ****P<0.0001 (determined using ANOVA). ns, non-significant.

Using tail amputation in 3 days post fertilization (dpf) zebrafish larvae as a model for inflammation, we studied the effect of four glucocorticoids (beclomethasone, dexamethasone, hydrocortisone and prednisolone) on the migration of leukocytes towards a site of injury. To quantitate the migration of neutrophils and macrophages, we counted the number of these innate immune cells in a defined area of the tail at 4 h post amputation (hpa, Fig. 1A). All four glucocorticoids had a highly significant inhibitory effect on the migration of neutrophils, as previously observed (Hall et al., 2014; Fig. 1C). Three glucocorticoids (beclomethasone, dexamethasone and prednisolone) did not affect the migration of macrophages significantly, and one (hydrocortisone) induced a slight decrease (∼12.5%, Fig. 1B). These data are in line with a previous study from our group, in which we demonstrated that beclomethasone inhibited the migration of neutrophils and not of macrophages, that this effect was mediated through Gr, and that beclomethasone did not affect the total leukocyte numbers in the larvae (Chatzopoulou et al., 2016).

**Fig. 2.**
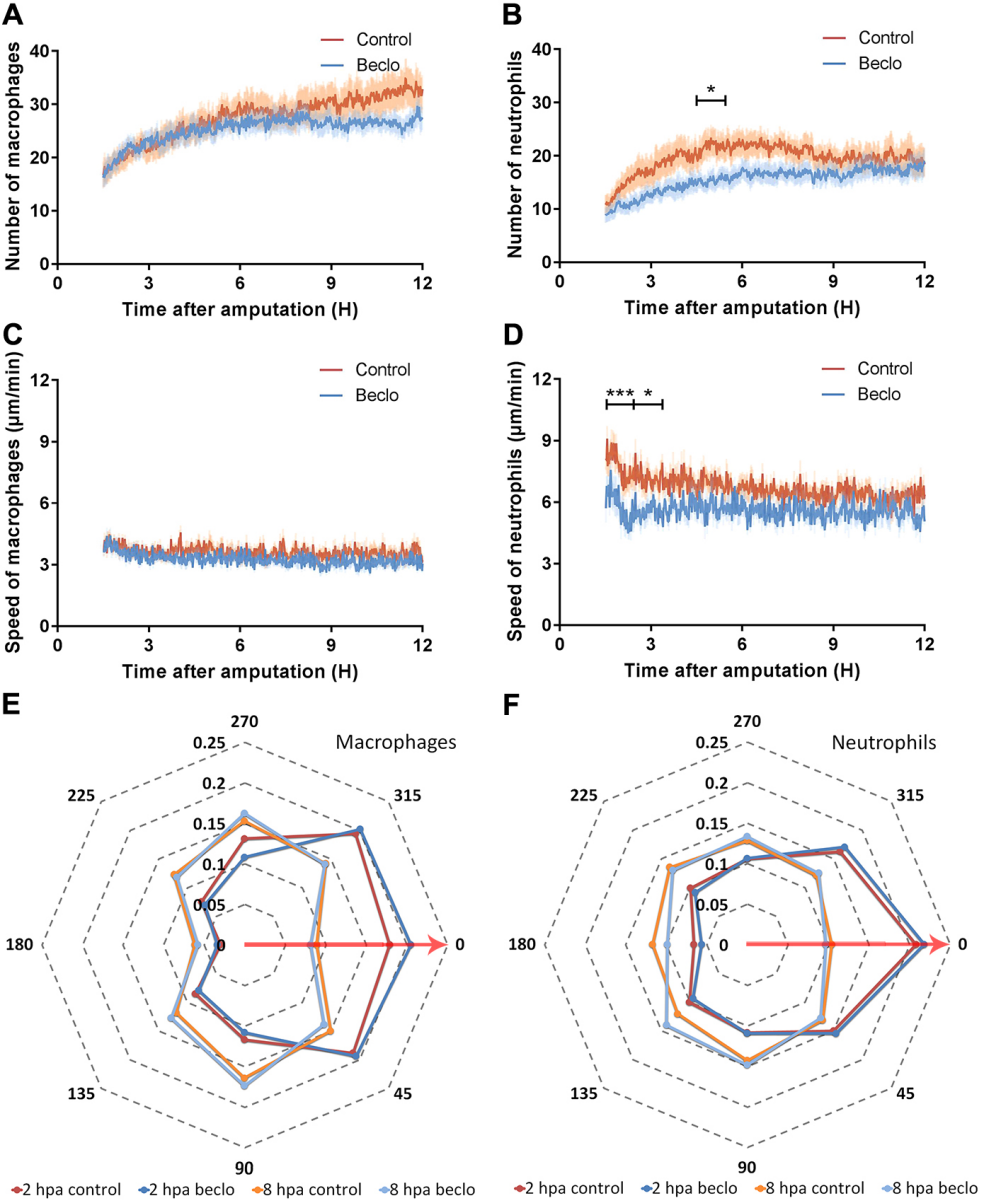
**Live imaging and tracking of migrating macrophages and neutrophils upon tail amputation.** (A-B) The number of macrophages (A) and neutrophils (B) recruited to the wounded area from 1.5 hpa to 12 hpa in 3 dpf larvae in the vehicle-treated group (Control) and the beclomethasone-treated group (Beclo). No significant difference was observed for the number of recruited macrophages. A significantly reduced number of neutrophils were recruited in the beclomethasone-treated group compared to the control group at 5 hpa. Data are mean±s.e.m. from 10 larvae. *P<0.05 (determined on data averaged per hour using ANOVA with a Fisher's LSD post hoc test). (C-D) The velocity of macrophages (C) and neutrophils (D). No significant difference was observed for the velocity of macrophages. At 2 and 3 hpa, the velocity of neutrophils in the beclomethasone-treated group was significantly lower than the velocity in the control group. Data are mean±s.e.m. from 10 embryos. *P<0.05; ***P<0.001 (determined on data averaged per hour using ANOVA with a Fisher's LSD post hoc test). (E-F) The directionality of recruited macrophages (E) and neutrophils (F) at 2 hpa and 8 hpa. The circular *x*-axis indicates the different angles made by cells, classified into eight categories. Category 0 represents the direction towards the wound (including angles between 22.5 to −22.5°, shown by the red arrows). The *y*-axis indicates the size of the fraction of cells occurring within a category in that hour. Statistical analysis was performed using the Kolmogorov–Smirnov test. No difference was observed between the control and beclomethasone-treated groups. Data are mean from 10 embryos.

To study the effects of beclomethasone on leukocyte migration in more detail, larvae were imaged using confocal microscopy between 1.5 and 12 hpa, and the leukocyte numbers in the wounded area were automatically determined using dedicated software. The results of this analysis showed that, for the control group, the average number of macrophages present in the wounded area increased from 37.0±3.5 to 48.7±4.1 cells between 1.5 hpa and 12 hpa (Fig. 2A; data are mean±s.e.m.). No significant effect of beclomethasone on macrophage migration was observed (from 37.6±2.8 to 41.4±2.5 for the beclomethasone-treated group). For neutrophils, in the control group, the average number of macrophages at 1.5 hpa was 17.7±2.0, reaching a peak of 35.1±4.1 at around 5 hpa, then decreasing and reaching a level of 32.7±3.3 at 9 hpa, which remained relatively constant until 12 hpa (Fig. 2B). In the beclomethasone-treated group, a lower number of recruited neutrophils was observed in the wounded area at 5 hpa (22.8±1.9).

To further analyze the effects of beclomethasone, we used automated tracking of the leukocytes (see Movies 1-2), and quantified the velocity and directionality of the migrating macrophages and neutrophils. The data showed that, during the entire time frame, the velocity of the macrophages fluctuated around 3.5 µm/min for both the control and the beclomethasone-treated group (Fig. 2C). For neutrophils, the velocity peaked at 1.5 hpa (8.12±0.56 µm/min for the control group and 5.70±0.72 µm/min for the beclomethasone-treated group) and decreased slowly afterwards (Fig. 2D). At 2 hpa and 3 hpa, the velocity of neutrophils in the beclomethasone-treated group was significantly lower compared to the control group.

In addition, we measured the direction in which the macrophages and neutrophils moved and plotted the distribution of these directions measured at 2 and 8 hpa (Fig. 2E,F). The results showed that beclomethasone did not affect the directionality of either macrophages or neutrophils at either of these time points. At 2 hpa, most of the macrophages (∼60%) moved towards the wounded area (angles 292.5°-360°, and 0°-67.5°) (Fig. 2E); less than 20% of them moved in the opposite direction (angles 112.5°-247.5°). At 8 hpa, the percentage of macrophages that moved towards the wounded area in the control and beclomethasone-treated group decreased to ∼40%. For the neutrophils, the directionality showed a similar trend (Fig. 2F). At 2 hpa, more than 50% of the neutrophils moved towards the wounded area in both the control group and the beclomethasone-treated group, whereas at 8 hpa this percentage decreased to ∼35%. In conclusion, beclomethasone does not affect any of the migration parameters of macrophages but reduces the number of recruited neutrophils and their velocity.

### Beclomethasone inhibits the induction of chemoattractants for macrophages

**Fig. 3.**
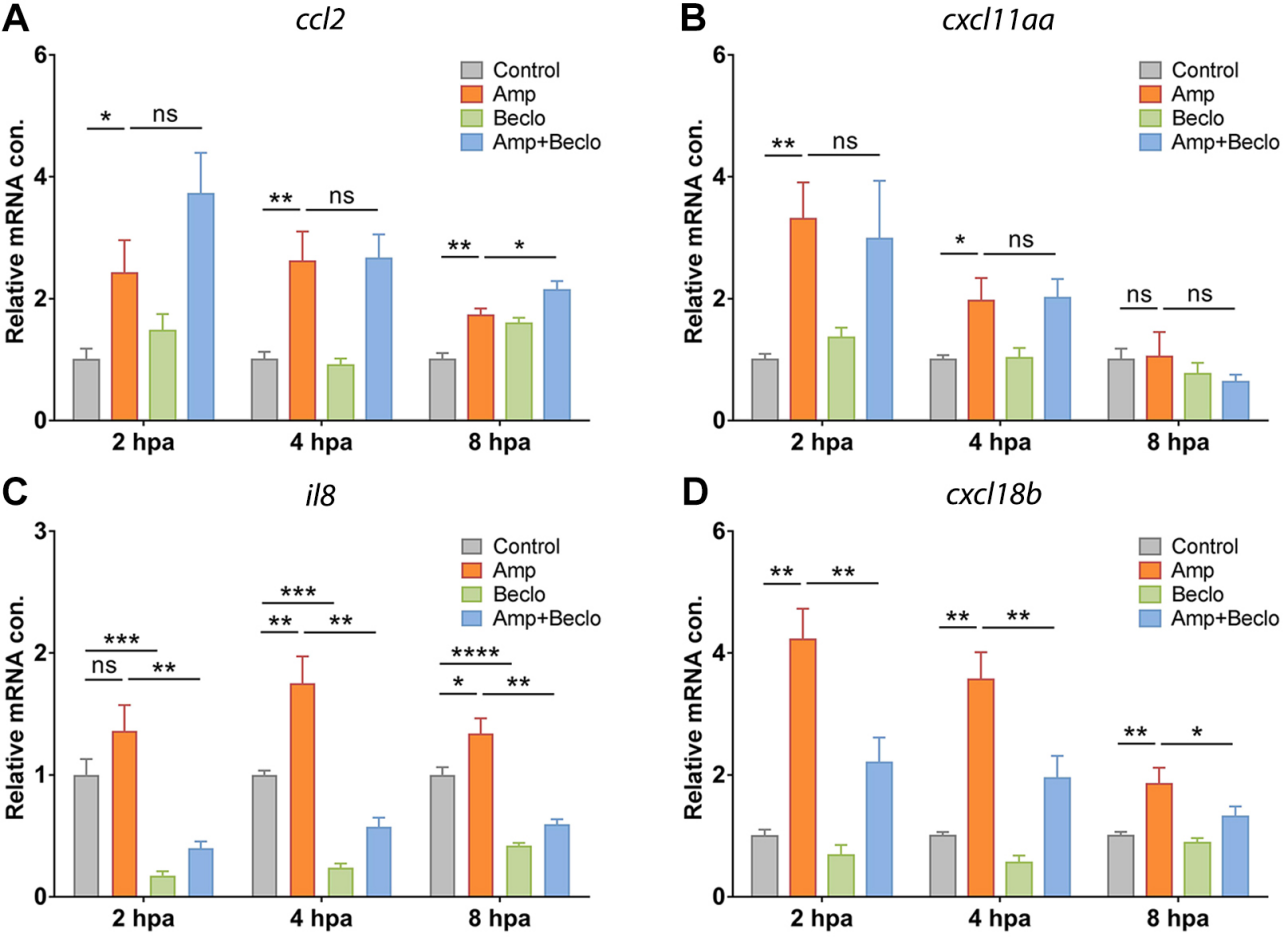
**Expression levels of genes encoding chemoattractants in whole larvae.** (A-D) Expression levels of genes encoding chemoattractants Ccl2 (A), Cxcl11aa (B), Il8 (C) and Cxcl18b (D) in whole larvae at 2 hpa, 4 hpa and 8 hpa, determined using qPCR. *ccl2* and *cxcl11aa* mRNA levels were significantly increased by amputation (Amp) and the combined amputation/beclomethasone (Amp+Beclo) treatment resulted in a similar level of regulation, relative to the non-amputated, vehicle-treated group (Control). Expression levels of *il8* and *cxcl18b* showed a significant increase upon amputation, and this effect was lower upon the combined treatment. Expression level of *il8* was significantly suppressed by beclomethasone (Beclo). Data are mean±s.e.m. of three independent experiments. *P<0.05; **P<0.01; ***P<0.001 (determined using ANOVA with a Fisher's LSD post hoc test). ns, non-significant.

To unravel the molecular mechanisms underlying the difference between the effect of beclomethasone on macrophage and neutrophil migration, we first studied the expression of chemoattractants that are known to be involved in the migration of these leukocytes. According to previous studies on leukocyte migration and infiltration, Ccl2 (also known as monocyte chemoattractant protein 1, Mcp1) and Cxcl-11aa (Cxcl11.1) are two of the key chemokines that stimulate the migration of macrophages, whereas Il8 (Cxcl8a) and Cxcl18b (Cxcl-c1c) are important for the stimulation of neutrophil migration (Cambier et al., 2017; de Oliveira et al., 2013; de Oliveira et al., 2016; Deshmane et al., 2009; Huber et al., 1991; Torraca et al., 2015; Torraca et al., 2017). Using quantitative PCR (qPCR) on RNA samples from whole larvae, we determined the expression levels of the genes encoding these four chemoattractants (*ccl2*, *cxcl11aa*, *il8* and *cxcl18b*) at different time points after amputation (Fig. 3A-D). The results showed that, at 4 hpa, the mRNA level of all four chemoattractants was increased by amputation. At 2 hpa, the expression of *ccl2*, *cxcl11aa* and *cxcl18b* was increased, and at 8 hpa the expression of *ccl2*, *il8* and *cxcl18b* showed an increase. In the presence of beclomethasone, amputation induced a smaller increase in *il8* and *cxcl18b* expression, but the increase in expression of *ccl2* and *cxcl11aa* was not inhibited. In addition, beclomethasone decreased the expression of *il8* independent of amputation. We previously observed a similar suppression under basal conditions by beclomethasone for *mmp9*, *mmp13* and *il1b* (Chatzopoulou et al., 2016), indicating that for some immune-related genes, glucocorticoids downregulate the basal expression, in addition to attenuating their upregulation.

**Fig. 4.**
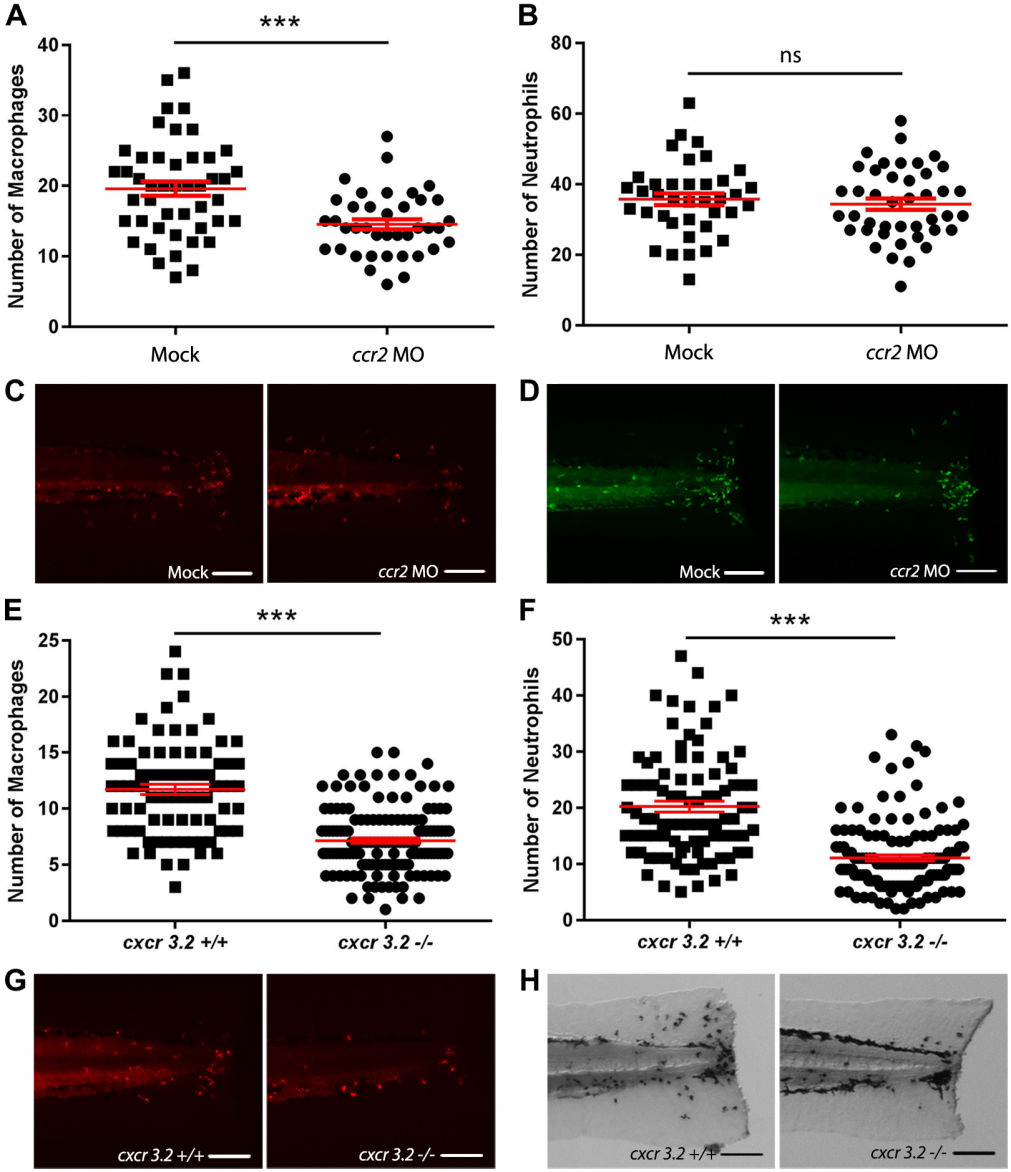
**Effect of *ccr2* morpholino knockdown or *cxcr3.2* mutation on macrophage and neutrophil recruitment upon tail amputation in larvae.** (A-B) The number of macrophages (A) and neutrophils (B) recruited to the wounded area at 4 hpa in 3dpf *Tg*(*mpx:GFP/mpeg1:mCherry-F*) larvae. In *ccr2* morpholino-injected larvae, a significantly reduced number of macrophages were recruited compared to the number in mock (vehicle)-injected larvae. No significant difference was observed for the number of recruited neutrophils. (C-D) Representative images of the macrophages (fluorescently labeled by mCherry) (C) and the neutrophils (fluorescently labeled by GFP) (D) of mock-injected and *ccr2* morpholino-injected larvae at 4 hpa. (E-F) The number of macrophages (E) and neutrophils (F) that recruited to the wounded area at 4 hpa in 3 dpf *Tg*(*mpeg1:mCherry-F*) larvae. A significantly reduced number of macrophages and neutrophils were recruited in *cxcr3.2* mutant larvae compared to the number in wt controls. (G-H) Representative images of the macrophages (fluorescently labeled by mCherry) (G) and the neutrophils (stained using MPX assay) (H) of wt and *cxcr3.2* mutant larvae at 4 hpa. Data are mean±s.e.m. (indicated in red), pooled from three independent experiments. ***P<0.001 (determined using the two-tailed t-test). ns, non-significant. Scale bars: 100 μm.

To demonstrate that the chemoattractants Ccl2 and Cxcl11aa are required for macrophage recruitment in this tail amputation model, we analyzed their role in macrophage migration in our model. We used a previously described morpholino to create a knockdown of Ccr2, the receptor of Ccl2, in zebrafish larvae, which was shown not to affect the total number of leukocytes (Cambier et al., 2017; Cambier et al., 2014). In the *ccr2* morphants, a significantly decreased number of recruited macrophages was observed in the wounded area at 4 hpa (Fig. 4A,C). However, the number of recruited neutrophils was identical to the number in the mock-injected controls (Fig. 4B,D) [the number of recruited neutrophils was unexpectedly high in these experiments (compared to data shown in Figs 1C, 2B and 4F), which we can only explain as an effect of the injections]. For the receptor of Cxcl11aa, Cxcr3.2, we used a mutant fish line, and it was previously demonstrated that total numbers of leukocytes were not affected by the mutation (Torraca et al., 2015). The *cxcr3.*2^−/−^ larvae showed significantly decreased numbers of both macrophages (Fig. 4E,G) and neutrophils (Fig. 4F,H) recruited to the wounded area compared to the *cxcr3.2*^+/+^ controls [the number of recruited macrophages was slightly lower in these experiments (compared to data shown in Figs 1B, 2A and 4A), which may be because of the different genetic background of the used fish line].

These findings indicate that beclomethasone does not affect the amputation-induced increase in the expression of the genes encoding the chemoattractants Ccl2 and Cxcl11aa, which are involved in macrophage recruitment upon tail amputation. This provides an explanation for the insensitivity of macrophage migration to glucocorticoid treatment.

### Beclomethasone attenuates almost all amputation-induced changes in gene expression in macrophages

**Fig. 5.**
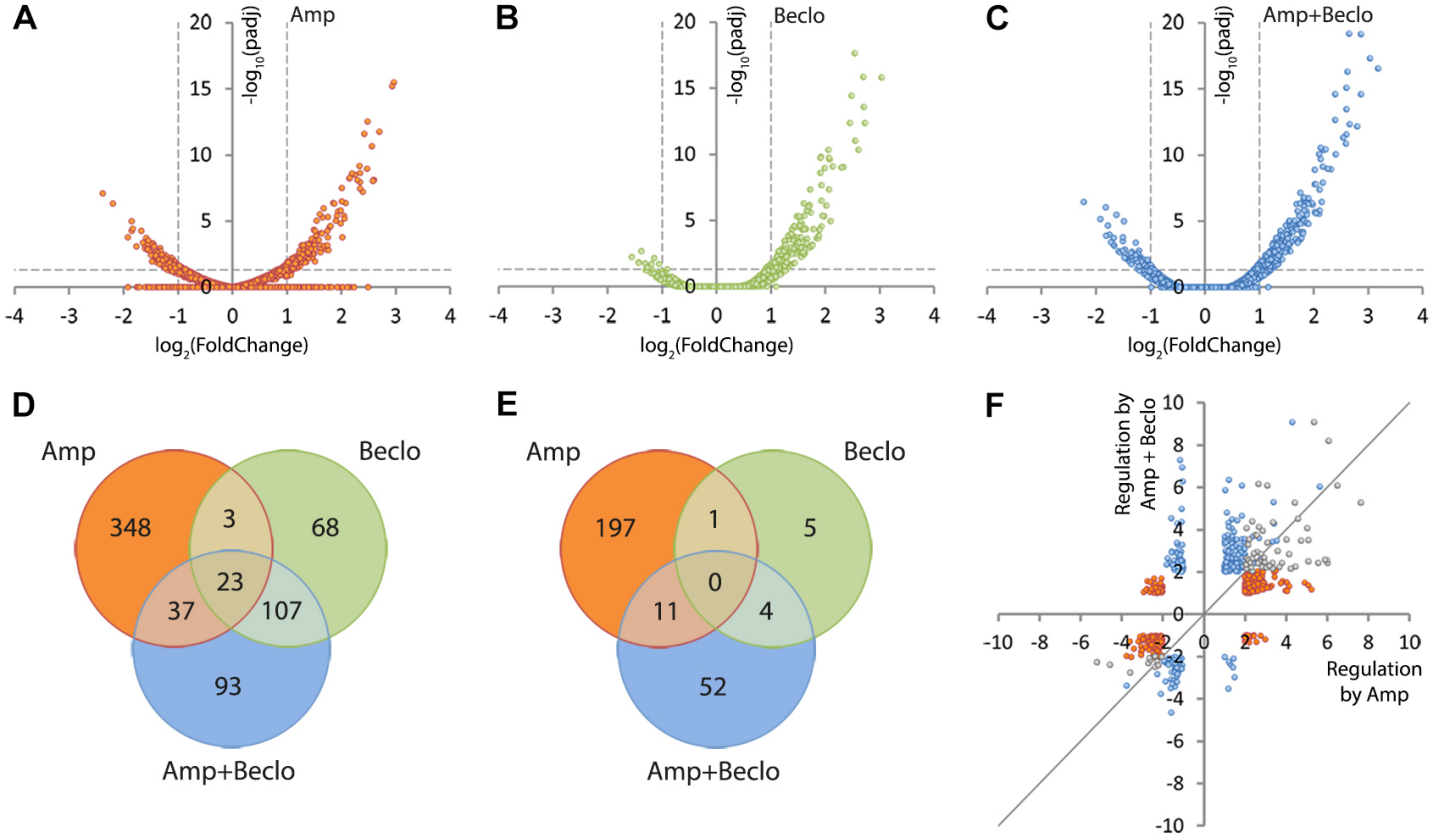
**Macrophage-specific transcriptome analysis by RNA-seq showing modulation of amputation-induced gene regulation by beclomethasone.** (A-C) Volcano plots indicating the fold change (*x*-axis) and *P*-value (*y*-axis) of the regulation for individual genes by amputation (A), beclomethasone (B) and the combined amputation/beclomethasone treatment (C), compared to the non-amputated, vehicle-treated control group. (D-E) Venn diagrams showing overlaps between clusters of genes significantly upregulated (D) or downregulated (E) by amputation (Amp), beclomethasone (Beclo) and the combined amputation/beclomethasone treatment (Amp+Beclo). The diagrams show that there is a large number of genes regulated by amputation in macrophages. Beclomethasone affects the expression of a relatively small number of genes, but it decreases the number of genes significantly regulated upon amputation. (F) Scatter plot showing the effect of beclomethasone treatment on amputation-regulated gene expression. For all genes showing significant regulation upon amputation (red and gray dots) or the combined beclomethasone and amputation treatment (blue and gray dots), the fold change due to beclomethasone and amputation treatment was plotted as a function of the fold change due to amputation (gray dots represent the overlap between amputation and combination treatment). The gray line indicates the point at which beclomethasone treatment does not alter amputation-induced gene regulation. Of all the genes that were significantly regulated by amputation in macrophages, 75.37% showed attenuation in the presence of beclomethasone. Paired analysis were performed using DESeq (v1.26.0) R package by comparing each group to the control group (non-amputated/vehicle treated). Significantly regulated genes were selected by using a p.adj<0.05 and |FoldChange|>2 cutoff.

To study whether glucocorticoid treatment changes the transcriptional response of macrophages to wounding, we performed a transcriptome analysis on fluorescence-activated cell sorting (FACS)-sorted macrophages derived from larvae at 4 hpa. We found that 620 genes were significantly regulated by amputation, of which 411 genes were upregulated and 209 genes were downregulated (Fig. 5A,D,E). When the larvae had been amputated and treated with beclomethasone, only 327 significantly regulated genes were identified, of which 260 genes were upregulated and 67 genes were downregulated (Fig. 5B,D,E). Apparently, amputation-induced gene regulation in macrophages is attenuated by beclomethasone administration. To study the effect of beclomethasone on the amputation-induced changes in gene expression in macrophages in more detail, we plotted the level of regulation by the combined amputation and beclomethasone treatment against the regulation by amputation in the absence of beclomethasone for all genes that were significantly regulated by at least one of these treatments (Fig. 5F). The resulting scatter plot shows that 75.37% of the genes regulated by amputation showed attenuation of this regulation when amputation was performed in the presence of beclomethasone. These results indicate that beclomethasone has a very general and strong dampening effect on the amputation-induced changes in gene expression in macrophages, which is in contrast with the lack of inhibition of the migration of these cells towards the wounded area.

Interestingly, only a small overlap was observed between the cluster of 620 amputation-regulated genes and the cluster of 327 genes regulated by the combined amputation and beclomethasone treatment (Fig. 5A,C,D,E). Only 60 and 11 genes were present in the overlap between these clusters for upregulation and downregulation, respectively (Fig. 5D,E). A large overlap was observed between the gene cluster regulated by the combination treatment and the cluster regulated by beclomethasone (without amputation) (134 genes in total, Fig. 5B,C,D,E, Fig. S1A). This indicates that the cluster of genes regulated by the combination treatment mainly contains genes that are regulated as a result of the beclomethasone treatment. Apparently, amputation hardly affects beclomethasone-induced changes in gene expression, whereas beclomethasone has a very strong effect on amputation-induced transcriptional changes. The smallest overlap was observed between the cluster of amputation-regulated genes and the cluster of beclomethasone-regulated genes (Fig. 5A,B,D,E, Fig. S1B), which suggests that, upon amputation, endogenous glucocorticoid signaling due to increased cortisol levels only regulates a small number of genes.

Using gene ontology analysis, we classified the regulated genes according to the KEGG pathways that they are involved in (Fig. S2, Table S1). This analysis showed that the largest group of pathways regulated by amputation were involved in metabolism (16 pathways, 98 genes) and that four pathways (19 genes) involved in the immune system were altered. The combined amputation and beclomethasone treatment affected a smaller number of pathways for both metabolism- and immune system-related pathways (12 pathways and 26 genes, and one pathway and six genes, respectively). Only five of these pathways (Toll-like receptor signaling pathway, Insulin resistance, Biosynthesis of antibiotics, Galactose metabolism, Glycolysis/Gluconeogenesis) were both regulated by amputation and by the combination treatment. Beclomethasone treatment (without amputation) affected seven pathways (five metabolism-related), of which six were also regulated when the larvae were amputated in the presence of beclomethasone.

Among the significantly enriched metabolism-related KEGG pathways, we studied three specific pathways which are known to be associated with specific macrophage phenotypes: glycolytic metabolism, which is increased in pro-inflammatory (M1) macrophages, and mitochondrial oxidative phosphorylation (OXPHOS) and the tricarboxylic acid (TCA) cycle, which are related to the anti-inflammatory (M2) phenotype (Van den Bossche et al., 2015). We mapped the gene expression levels into these pathways (Fig. S3A-C). The data showed that the vast majority of the mapped genes were upregulated by amputation and this upregulation was inhibited by beclomethasone treatment. We, therefore, conclude from the gene ontology analysis that amputation mainly upregulates genes involved in metabolism and the immune system, and that the vast majority of the amputation-induced changes in these gene ontology groups are attenuated by glucocorticoids.

### Glucocorticoids inhibit the differentiation of macrophages towards a pro-inflammatory phenotype

**Fig. 6.**
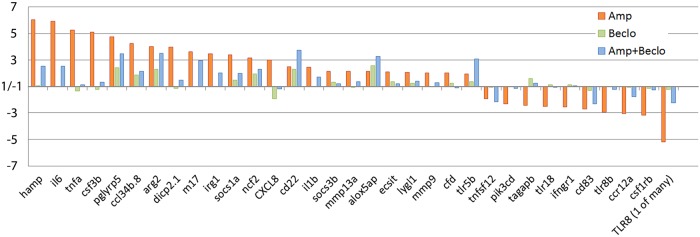
**Regulation of immune-related genes in macrophages, determined by RNA-seq analysis.** For all genes significantly regulated upon amputation, the fold change due to amputation (Amp; red bars), beclomethasone (Beclo; green bars) and the combined amputation/beclomethasone treatment (Amp+Beclo; blue bars) is shown. The results show that beclomethasone dampens the amputation-induced expression of most genes, but for three genes (*cd22*, *alox5ap*, *tlr5b*) the combined treatment results in a higher fold change compared to the amputation treatment. Paired analysis was performed using DESeq (v1.26.0) R package by comparing each group to the control group (non-amputated/vehicle treated). Significantly regulated genes were selected by using a p.adj<0.05 and |FoldChange|>2 cutoff.

Subsequently, we specifically analyzed the regulation of immune-related genes. For all immune-related genes that were significantly regulated by amputation, we plotted the regulation by amputation, by beclomethasone, and by the combination of amputation and beclomethasone (Fig. 6). For the vast majority of these genes, the amputation-induced changes were attenuated by the administration of beclomethasone. Among those genes were three that are known to be associated with a pro-inflammatory (M1) phenotype of macrophages: *tnfa*, *il1b* and *il6* (Martinez and Gordon, 2014; Nguyen-Chi et al., 2015). For three genes (*cd22*, *alox5ap* and *tlr5b*), the amputation-induced regulation was enhanced by beclomethasone. These findings suggest that the differentiation of macrophages to a pro-inflammatory (M1) phenotype is sensitive to inhibition by glucocorticoids.

**Fig. 7.**
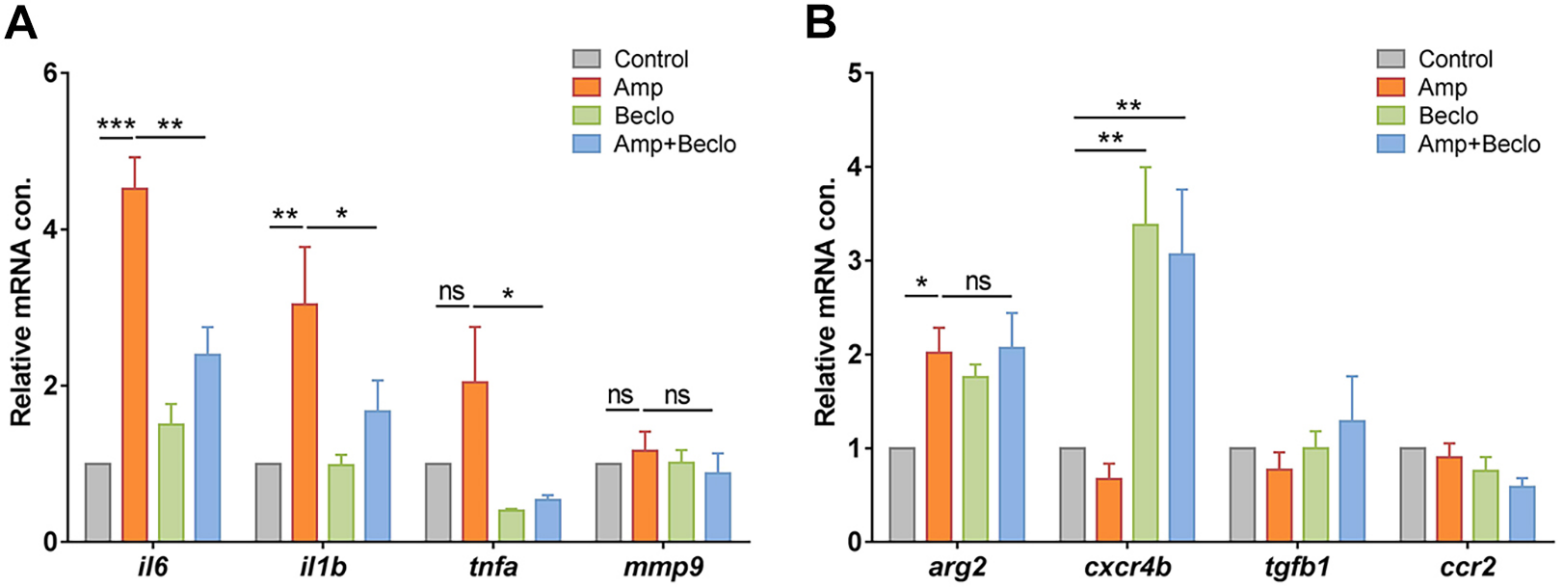
**Expression levels of immune-related genes in FACS-sorted macrophages.** (A-B) Expression levels of immune-related genes in FACS-sorted macrophages, determined by qPCR for *il6*, *il1b*, *tnfa*, *mmp9* (A) and for *arg2*, *cxcr4b*, *tgfb1*, *ccr2* (B) at 4 hpa in 3 dpf larvae. Statistical analysis showed that the levels of *il6* and *il1b* expression were significantly increased by amputation, and this effect was inhibited by beclomethasone treatment. The expression level of *arg2* showed a significant increase upon amputation, and beclomethasone treatment did not affect this regulation. The expression level of *cxcr4b* was increased by beclomethasone treatment. Data are mean±s.e.m. of three independent experiments. *P<0.05; **P<0.01; ***P<0.001 (determined using ANOVA with a Fisher's LSD post hoc). ns, non-significant.

To study the glucocorticoid sensitivity of macrophage differentiation in more detail and validate some of the observed transcriptional changes, we performed qPCR on RNA samples isolated from FACS-sorted macrophages. At 4 hpa, the expression of four classic pro-inflammatory genes was measured: *il6*, *il1b*, *tnfa* and *mmp9*, of which the first three are markers for M1 macrophages and the fourth encodes a metalloproteinase that facilitates leukocyte migration by remodeling the extracellular matrix (Martinez and Gordon, 2014; Nguyen-Chi et al., 2015; Rohani and Parks, 2015) (Fig. 7A). The expression levels of *il6* and *il1b* showed an amputation-induced increase, and this increase was attenuated upon the combined beclomethasone and amputation treatment. The levels of *tnfa* and *mmp9* expression were not significantly increased by amputation, but the expression level of *tnfa* was significantly lower after the combination treatment compared to the amputation treatment.

In addition, we measured the expression levels of four markers for M2 macrophages, *arg2*, *cxcr4b*, *tgfb1* and *ccr2* (Nguyen-Chi et al., 2015; Yang and Ming, 2014) (Fig. 7B). The expression level of *arg2* was increased by amputation at 4 hpa, and this was similar upon the combination treatment. The other genes were not upregulated by amputation at this time point, but upon beclomethasone treatment the expression of *cxcr4b* was increased. As the M2 macrophage markers are expected to show increased expression levels during the resolution phase of the inflammatory response (Nguyen-Chi et al., 2015), we measured the expression of those genes in macrophages at 24 hpa as well (Fig. S4A). However, no significant upregulation by amputation was observed for any of these four genes. Thus, in this experiment on M2 markers, we only found an amputation-induced upregulation of the expression of *arg2* at 4 hpa, and this upregulation was insensitive to beclomethasone.

**Fig. 8.**
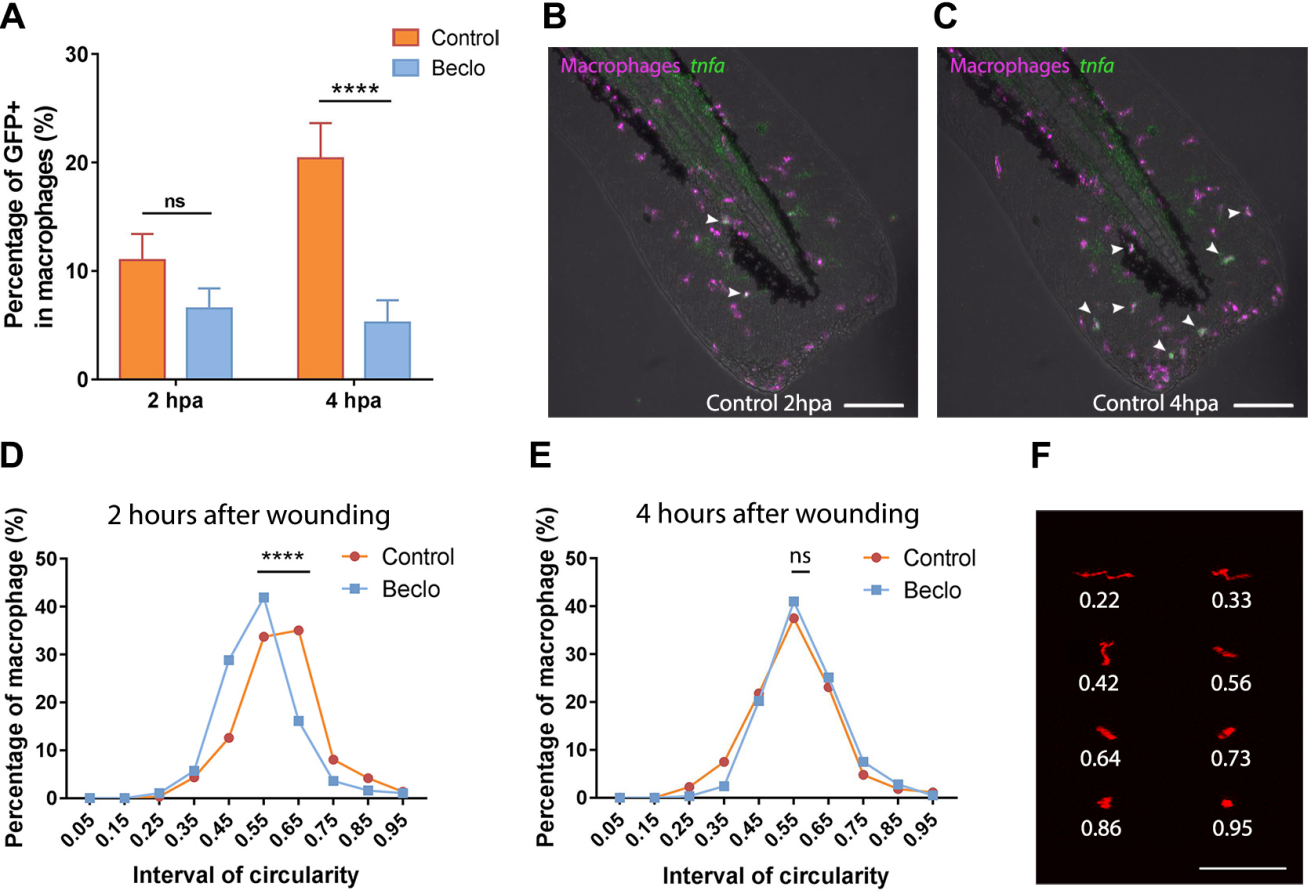
**Effect of beclomethasone on the phenotype of macrophages.** (A) In *Tg(mpeg1:mCherry-F/tnfa:eGFP-F)* reporter larvae, the number of GFP-positive macrophages (as a percentage of the total number of macrophages) recruited to the wounded area was quantified at 2 and 4 hpa in 5 dpf larvae. In the beclomethasone-treated group (Beclo), at 4 hpa, a significantly reduced percentage of the recruited macrophages was GFP-positive compared to the vehicle-treated group (Control). Data are mean±s.e.m. ****P<0.0001 (determined using ANOVA with a Fisher's LSD post hoc). (B-C) Representative images of macrophages (fluorescently labeled by mCherry) and GFP-positive macrophages (fluorescently labeled by both mCherry and GFP) in the control group at 2 hpa (B) and 4 hpa (C). Arrowheads indicate macrophages displaying the GFP signal, which is a measure for activation of the *tnfa* promoter. (D-E) The distribution of circularity of macrophages recruited to the wounded area at 2 h after wounding (hpa) (D) and at 4 h after wounding (E) in 3 dpf *Tg(mpeg1:mCherry-F)* larvae. At 2 hpa, a significant difference of distribution pattern was observed between the two groups, with the beclomethasone-treated group (Beclo) shifted towards lower circularity. At 4 hpa, no significant difference was observed. ****P<0.0001 (determined using the Kolmogorov–Smirnov test). (F) Representative images of macrophages analyzed in D and E and their corresponding circularity. ns, non-significant. Scale bars: 100 μm.

To further study the influence of beclomethasone on the differentiation of macrophages towards a pro-inflammatory (M1) phenotype, we used a reporter line for the expression of *tnfa*: the *Tg(mpeg1:mCherry-F/tnfa:eGFP-F)* fish line. Larvae from this line were amputated at 5 dpf, and at a more distal position than in the previous experiments to create a wound that recruits fewer macrophages, which facilitates the visualization of individual *tnfa*-expressing macrophages. We performed live confocal imaging at 2 and 4 hpa, and the GFP expression level in macrophages was used as a reporter for *tnfa* promoter activity *in vivo* (Fig. 8A-C). In the control group, an increase in the percentage of GFP-positive macrophages was observed between 2 and 4 hpa, from 9.8±3.4% to 23.8±4.0%. The images show that GFP expression does not exclusively occur in macrophages that have reached the wounded area. In the beclomethasone-treated group, at both time points, a lower percentage of *tnfa*-expressing macrophages was recruited to the wounded area compared to the control group (1.7±1.7% and 1.4±1.4% for 2 and 4 hpa, respectively).

Finally, we analyzed the influence of beclomethasone on the morphology of macrophages, as macrophage morphology has been shown to be an indicator for their differentiation: M1 macrophages are generally less elongated and dendritic than M2 macrophages (Nguyen-Chi et al., 2015). Instead of amputation, a small hole was punched in the tail fins of the larvae using a glass microcapillary needle to recruit a reduced number of leukocytes, which facilitated the visualization of individual cells. We performed live confocal imaging at 3 dpf with the *Tg(mpx:GFP/mpeg1:mCherry-F)* fish line and the circularity of mCherry-positive macrophages was used to quantitate the morphology (Fig. 8D,E). In the control group, at 2 hpa, the percentage of macrophages with a high circularity (0.5-1.0) was relatively high (67.6±4.0%) and gradually decreased to 47.9±3.2% at 12 hpa (Fig. S5A). In the beclomethasone-treated group, at 2 hpa the percentage of macrophages with a high circularity was lower (51.7±3.5%) and remained relatively stable until 12 hpa (Fig. S5B). The most obvious difference between the control and beclomethasone-treated group was observed at 2 hpa. At this time point, the plot showing the distribution of circularity shows a clear shift towards a lower circularity in the beclomethasone-treated group (Fig. 8D,F). At 4 hpa, this difference of circularity distribution between the control group and beclomethasone-treated group had disappeared (Fig. 8E,F). The highly transient nature of the increased circularity is probably because of the small size of the wound in this experiment. These data from the analysis of the circularity may suggest an inhibitory effect of beclomethasone on the differentiation of macrophages towards a pro-inflammatory (M1) phenotype, in line with the data obtained using the *tnfa:eGFP-F* reporter line.

## DISCUSSION

Although glucocorticoids have been used as anti-inflammatory drugs for decades, their mechanism of action and the specificity of their effects have not been fully unraveled yet. Using the zebrafish tail amputation model, we have shown that the inflammatory response comprises glucocorticoid-sensitive and glucocorticoid-insensitive pathways. Glucocorticoids inhibit the migration of neutrophils towards a site of inflammation by inhibiting the induction of chemoattractants for this cell type. However, the migration of macrophages is not affected by glucocorticoids, as the induction of two chemoattractants that are critical for macrophage recruitment, *ccl2* and *cxcl11aa*, is insensitive to treatment with the glucocorticoid beclomethasone. Using RNA-seq analysis we show that beclomethasone attenuates most transcriptional responses to amputation in macrophages and inhibits their differentiation towards a pro-inflammatory (M1) phenotype.

Chemoattractants are important trafficking signals that direct the movement of immune cells into and out of specific tissues (Luster et al., 2005). In this study, we have demonstrated that glucocorticoids exert a specific inhibitory effect on the induction of the expression of two chemoattractants involved in neutrophil recruitment (Il8 and Cxcl18b). Using *in vitro* and *in vivo* models, it has been demonstrated that human and mouse neutrophil migration is dependent on the induction of Il8 expression (Godaly et al., 2000; Huber et al., 1991; Kaunisto et al., 2015) and that this induction is inhibited by glucocorticoids (Huang et al., 2015; Keelan et al., 1997; Yano et al., 2006). In mammals, Il8 has been demonstrated to signal through the chemokine receptors Cxcr1 and Cxcr2, whereas in zebrafish only Cxcr2 has been shown to mediate the effects of Il8 (Brugman, 2016; Torraca et al., 2015). Interestingly, our RNA-seq data show that amputation increased the expression of *il8* in macrophages, and that this increase was strongly attenuated by beclomethasone. These data suggest that the glucocorticoid inhibition of the neutrophil migration results at least partly from the suppression of chemoattractant expression in macrophages. Cxcl18b, a chemokine that is specific for fish and amphibian species, has also been shown to act as a ligand for Cxcr2 in zebrafish, thereby stimulating chemotaxis of neutrophils (Torraca et al., 2017). These findings suggest that Cxcr2 activation is crucial for the migration of neutrophils, and that glucocorticoids inhibit this migration by attenuating the induction of the expression of Cxcr2 agonists such as Il8 and Cxcl18b.

In contrast to the inhibitory effect on neutrophil migration, our study revealed that glucocorticoids do not affect the induction of chemoattractants involved in macrophage recruitment (Ccl2 and Cxcl11aa). Ccl2 and Cxcl11aa have been shown to be key chemokines implicated in macrophage migration and infiltration in humans and mice (Deshmane et al., 2009; Gunn et al., 1997; Lee et al., 2018; Shen et al., 2014; Szebeni et al., 2017; Wada et al., 1999). In zebrafish, their role as chemoattractants for macrophages has been demonstrated during mycobacterial infection (Cambier et al., 2017; Cambier et al., 2014; Torraca et al., 2015). Our data show that these two chemoattractants also promote macrophage migration in the tail amputation model and that beclomethasone has no effect on the amputation-induced increase in their expression levels. The RNA-seq analysis showed very low expression levels of *ccl2* and undetectable levels of *cxcl11aa* expression in macrophages, which suggests that the contribution of these cells to the increased expression of these chemokines is limited.

In line with our findings, it has been shown in bronchoalveolar lavage fluid of COPD patients that glucocorticoid treatment reduces neutrophil numbers, but that the number of macrophages was not decreased (Jen et al., 2012). Contrary to our findings, in most of the studies carried out in humans and rats, the inflammation-induced Ccl2 level has been found to be inhibited by glucocorticoids (Kim et al., 1995; Little et al., 2006; Wada et al., 1999), and this inhibition is related to a decreased p38 MAPK phosphorylation (Baldassare et al., 1999; Little et al., 2006). Similarly, glucocorticoids have been shown to inhibit Cxcl11 upregulation in fluticasone propionate-stimulated peripheral blood monocytes, and in IFN-γ- or LPS-stimulated RAW 264.7 macrophages, as well as in multiple tissues of endotoxemia mice (Ehrchen et al., 2007; Widney et al., 2000). Nevertheless, some studies do show an insensitivity of the mammalian Ccl2 or Cxcl11aa induction to glucocorticoid treatment. In a breast cancer cell line (T47D), glucocorticoid treatment has no effect on Il1-stimulated Ccl2 production (Kelly et al., 1997), and in A579 epithelial cells, IFNγ-induced Cxcl11 is insensitive to glucocorticoid treatment (O'Connell et al., 2015). These data suggest that the observed insensitivity of the *ccl2* and *cxcl11a* induction to glucocorticoids, which underlies the glucocorticoid insensitivity of macrophage migration, requires a specific context, which may involve factors such as the activating signal, the glucocorticoid treatment regime, or the cell type and tissue involved.

Although glucocorticoids did not affect the migration of macrophages in our study, they did have a big impact on the transcriptional changes in these cells upon amputation. We showed, using RNA-seq analysis in FACS-sorted macrophages that, similarly to our previous findings from a microarray analysis carried out on RNA that was isolated from whole larvae (Chatzopoulou et al., 2016), most of the amputation-induced transcriptional changes are decreased by beclomethasone, whereas a small subset of transcriptional responses is insensitive to glucocorticoid treatment. Focusing on the regulation of immune-related genes, we found that, in line with our previous findings in whole larvae (Chatzopoulou et al., 2016), beclomethasone suppressed the induction of almost all pro-inflammatory M1 associated genes, such as *il6*, *tnfa*, *il1b*, *il8* and *mmp9*. In line with these data, many genes involved in glycolysis, a metabolic pathway often associated with an M1 phenotype (Kelly and O'Neill, 2015; Saha et al., 2017; Van den Bossche et al., 2015), were upregulated upon amputation and this upregulation was mostly inhibited by beclomethasone. This inhibitory effect of glucocorticoids on the induction of pro-inflammatory genes in macrophages is in agreement with *in vitro* results obtained in LPS-stimulated primary mouse macrophages (Ogawa et al., 2005; Sacta et al., 2018; Uhlenhaut et al., 2013). In addition, *in vivo* data obtained in mouse models for arthritis and acute lung injury demonstrated an inhibitory effect of glucocorticoids on the differentiation of macrophages towards a pro-inflammatory M1 phenotype (Hofkens et al., 2013; Tu et al., 2017). In the present study, we observed a reduction in the number of macrophages with activation of a *tnfa:eGFP-F* reporter gene upon beclomethasone administration, and a morphology characterized by a low circularity, which demonstrates that the macrophage differentiation to an M1 phenotype was inhibited by the glucocorticoid treatment. Taken together, these data strongly support the idea that glucocorticoids inhibit the differentiation of macrophages to an M1 phenotype by interfering at the level of transcription.

This glucocorticoid effect on macrophages may have great clinical relevance, as this cell type has been identified as the main target for glucocorticoid action in several animal models for inflammatory diseases (Bhattacharyya et al., 2007; Kleiman et al., 2012; Vettorazzi et al., 2015). In murine models for contact allergy and septic shock it has been shown that the anti-inflammatory effect of glucocorticoids depends on the presence of GR in macrophages, suppressing the induction of pro-inflammatory mediators such as IL-1β (Kleiman et al., 2012; Tuckermann et al., 2007). These glucocorticoid effects are absent in a mouse line with a deficiency in GR dimerization, suggesting that activation of anti-inflammatory gene transcription through GRE binding may be the main GR mechanism of action (Kleiman et al., 2012; Tuckermann et al., 2007). Furthermore, we conclude that the glucocorticoid resistance observed in macrophage-dominated inflammatory diseases such as COPD cannot be attributed to a general insensitivity of macrophages to the immune-suppressive effects of glucocorticoids.

In addition to the effect of glucocorticoids on M1 differentiation, we investigated their effect on the differentiation of macrophages to an M2 phenotype. Previous studies in a mouse arthritis model showed that the induction of an M2 phenotype was not affected by glucocorticoids (Hofkens et al., 2013) and in an acute lung injury model (Tu et al., 2017) it was shown to be enhanced. In our RNA-seq and qPCR analysis, the M2 marker *arg2* (Martinez and Gordon, 2014; Nguyen-Chi et al., 2015; Yang and Ming, 2014) was one of the small number of amputation-induced genes that was insensitive to beclomethasone, suggesting that the differentiation to an M2 phenotype is insensitive to glucocorticoids. However, genes involved in the TCA cycle and OXPHOS, metabolic pathways associated with an M2 phenotype (Kelly and O'Neill, 2015; Saha et al., 2017; Van den Bossche et al., 2015), were upregulated upon amputation, and this upregulation was inhibited by beclomethasone, which would suggest that M2 differentiation is blocked by glucocorticoid treatment. In our qPCR analysis, we showed that the expression of various other M2 markers (*cxcr4b*, *tgfb1*, *ccr2*) was not increased upon amputation. The variation in responses of M2 markers to amputation and/or glucocorticoid treatment in our assay supports the idea that the M2 phenotype of macrophages may occur as various alternative differentiation states (Martinez and Gordon, 2014; Mosser and Edwards, 2008). Independent of the amputation, beclomethasone increased the expression of *cxcr4b* (and to a lesser extent *ccr2*), in line with previous observations that glucocorticoids induce the differentiation of human macrophages to an M2 phenotype *in vitro* (Ehrchen et al., 2007; Heideveld et al., 2018). In summary, whereas the amputation-induced increases in the expression levels of M1 markers are consistently inhibited by beclomethasone, increased expression of M2 markers (when present in our assay) can be either insensitive to or suppressed by glucocorticoid treatment.

In our tail amputation model for inflammation, the vast majority of macrophage transcriptional responses were suppressed by glucocorticoids, and only a small subset of these responses was not affected. Studies in murine models for inflammatory diseases suggest that the anti-inflammatory GR action in macrophages depends on GRE-dependent transcriptional regulation, probably reducing the activation of a subset of pro-inflammatory transcription factors (Bhattacharyya et al., 2007; Kleiman et al., 2012; Tuckermann et al., 2007). Alternatively, our data may indicate an important role for GR interaction (‘tethering’) with the transcription factor NF-κB, as in many studies it has been shown that the NF-κB-mediated transcriptional activation can be suppressed by GR or remains unaffected (Kuznetsova et al., 2015; Ogawa et al., 2005; Rao et al., 2011; Sacta et al., 2018). Recruitment of IRF3 to the transcription initiation complex has been shown to be associated with sensitivity to GR suppression (Ogawa et al., 2005; Uhlenhaut et al., 2013).

In conclusion, our *in vivo* study of the glucocorticoid modulation of the transcriptional responses to wounding using the zebrafish tail amputation model shows that the vast majority of these responses are sensitive to glucocorticoids, and only a small subset is insensitive. These insensitive responses play a role in the migration of macrophages and possibly their differentiation to an M2 phenotype, whereas the sensitive responses are involved in the migration of neutrophils and the differentiation of macrophages to an M1 phenotype. Our data demonstrate that these processes can be regulated independently, and that glucocorticoids exert their immunosuppressive effects on macrophages by modulating differentiation rather than migration.

## MATERIALS AND METHODS

### Zebrafish lines and maintenance

Zebrafish were maintained and handled according to the guidelines from the Zebrafish Model Organism Database (http://zfin.org) and in compliance with the directives of the local animal welfare committee of Leiden University. They were exposed to a 14 h light and 10 h dark cycle to maintain circadian rhythmicity. Fertilization was performed by natural spawning at the beginning of the light period. Eggs were collected and raised at 28°C in egg water (60 µg/ml Instant Ocean sea salts and 0.0025% methylene blue).

The following fish lines were used in this work: wild-type (wt) strain AB/TL, the double transgenic lines *Tg(mpx:GFP/mpeg1:mCherry-F)* (Bernut et al., 2014; Renshaw et al., 2006) and *Tg(mpeg1:mCherry-F/tnfa:eGFP-F)* (Nguyen-Chi et al., 2015), and the combination of *Tg(mpeg1:mCherry-F)* and the homozygous mutants (*cxcr3.2*^−/−^) or wt siblings (*cxcr3.2*^+/+^) of the *cxcr3.2^hu6044^* mutant strain (Torraca et al., 2015).

### Tail amputation and chemical treatments

After anesthesia with 0.02% aminobenzoic acid ethyl ester (tricaine, Sigma Aldrich), the tails of 3 dpf embryos were partially amputated (Fig. 1A) with a 1 mm sapphire blade (World Precision Instruments) on 2% agarose-coated Petri dishes under a Leica M165C stereomicroscope (Chatzopoulou et al., 2016; Renshaw et al., 2006). In the experiment on larvae from the *Tg(mpeg1:mCherry-F/tnfa:eGFP-F)* line, the site of amputation was more distal, so the wound attracted a lower number of leukocytes, which facilitated the imaging of individual cells (Fig. 8A-C). In the experiment in which we determined the morphology of the macrophages (Fig. 8D-F), a hole was punched in the tail fin with a glass microcapillary needle (Harvard Apparatus, preparation of needles with 10-20 µm outer diameter described in Benard et al., 2012), in order to make an even smaller wound and attract an even lower number of leukocytes. Wounded and non-wounded (control) embryos were pretreated for 2 h with 25 μM beclomethasone (Sigma Aldrich) or vehicle [0.05% dimethyl sulfoxide (DMSO)] in egg water before amputation/wounding, and received the same treatment after the amputation/wounding.

### Imaging and image quantification

Images of fixed or live larvae were captured using a Leica M205FA fluorescence stereomicroscope, equipped with a Leica DFC 345FX camera. In all fish lines used, the macrophages were detected based on the fluorescence of their mCherry label. Neutrophils were detected based on either their fluorescent GFP label or their myeloperoxidase (mpx) staining. To quantify the number of macrophages and/or neutrophils recruited to the wounded area, the cells in a defined area of the tail (Fig. 1A) were counted manually. Data were pooled from two or three independent experiments, and the mean±s.e.m. of the pooled data are indicated.

### Confocal microscopy and image analysis

For time lapse imaging and automated tracking of the leukocyte migration, the amputated larvae were mounted in 1.2% low melting agarose in egg water containing 0.02% tricaine and 25 μM beclomethasone or 0.05% DMSO on 40 mm glass-bottom dishes (WillCo-dish, WillCo Wells) and covered with 1.5 ml egg water containing tricaine and beclomethasone or DMSO. Confocal microscopy was performed using a Nikon Eclipse Ti-E microscope with a Plan Apo 20X/0.75 NA objective. A 488 nm laser was used for excitation of GFP and a 561 nm laser was used for excitation of mCherry. Time-lapse microscopy was performed at 28°C with an interval of ∼1 min. From the obtained *z*-stacks, aligned maximum projection images were generated using NIS-Elements, which were further analyzed using ImageJ, with custom-made plugins developed by Dr Joost Willemse (Leiden University) for localizing and tracking cells [‘Local Maxima Stack’ and ‘Track Foci’, algorithms previously described in Celler et al. (2013)] and determining their circularity [calculated as (area×4π)/(circumference)^2^].

### Morpholino injection

A morpholino targeting the translational start site of the *ccr2* gene (5′AACTACTGTTTTGTGTCGCCGAC3′, purchased from Gene Tools) (Cambier et al., 2014) was prepared and stored according to the manufacturer's instructions. Injection of 1 nl (0.5 mM) of the morpholino solution was performed into the yolk of fertilized eggs at the 1-2 cell stage.

### RNA isolation, cDNA synthesis and qPCR

At different time points after amputation, larvae were collected (15-20 per sample) in QIAzol lysis reagent (Qiagen) for RNA isolation, which was performed using the miRNeasy mini kit (Qiagen), according to the manufacturer's instructions. Extracted total RNA was reverse-transcribed using the iScript™ cDNA Synthesis Kit (Bio-Rad). QPCR was performed on a MyiQ Single-Color Real-Time PCR Detection System (Bio-Rad) using iTaq™ Universal SYBR^®^ Green Supermix (Bio-Rad). The sequences of the primers used are provided in Table S2. Cycling conditions were pre-denaturation for 3 min at 95°C, followed by 40 cycles of denaturation for 15 s at 95°C, annealing for 30 s at 60°C, and elongation for 30 s at 72°C. Fluorescent signals were detected at the end of each cycle. Cycle threshold values (Ct values, i.e. the cycle numbers at which a threshold value of the fluorescence intensity was reached) were determined for each sample. For each sample, the Ct value was subtracted from the Ct value of a control sample, and the fold change of gene expression was calculated and adjusted to the expression levels of a reference gene [*peptidylprolyl isomerase Ab* (*ppiab*)]. Data shown are mean±s.e.m. of three independent experiments.

### Mpx staining

Larvae were fixed in 4% paraformaldehyde (PFA, Sigma Aldrich) at 4°C overnight, and rinsed with PBS containing 0.05% Tween 20. The mpx staining for the *cxcr3.2* mutant line was performed using the Peroxidase (Myeloperoxidase) Leukocyte kit (Sigma Aldrich), according to the manufacturer's instructions. To visualize both macrophages and neutrophils in the same larvae, the mpx staining was always performed after imaging of the fluorescent signal of the macrophages.

### FACS of macrophages

Macrophages were sorted from *Tg(mpeg1.4:mCherry-F)* embryos as previously described (Rougeot et al., 2014; Zakrzewska et al., 2010). Dissociation was performed using 100-150 embryos for each sample at 4 hpa using Liberase TL (Roche) and stopped by adding fetal calf serum to a final concentration of 10%. Isolated cells were resuspended in Dulbecco's PBS and filtered through a 40 μm cell strainer. Actinomycin D (Sigma Aldrich) was added (final concentration of 1 µg/ml) to each step to inhibit transcription. Macrophages were sorted based on their red fluorescent signal using a FACSAria III cell sorter (BD Biosciences). The sorted cells were collected in QIAzol lysis reagent (Qiagen) for RNA isolation. Extracted total RNA was either reverse-transcribed for qPCR or amplified using the SMART-seq V4 kit (Clontech) for sequencing.

### Transcriptome analysis

A total of 12 samples (four experimental groups obtained from three replicate experiments) were processed for transcriptome analysis using cDNA sequencing. The RNA-seq libraries generated with the SMART-seq V4 kit were sequenced using an Illumina HiSeq 2500 instrument according to the manufacturer's instructions, with a read length of 50 nucleotides. Image analysis and base calling were done using the Illumina HCS version 2.2.68, and RTA version 1.18.66. cDNA sequencing data were analyzed by mapping the reads to the *Danio rerio* GRCz10 reference genome with annotation version 80 using Tophat (v2.1.0). Subsequently, the DESeq (v1.26.0) R package was used to test for differential expression. Before each analysis, the genes with low reads were removed (i.e. those genes for which the sum of reads from three replicates of the analyzed two groups was lower than 30). The output data were used for transcriptome analysis. Significant gene regulation was defined by using p.adj<0.05 and |FoldChange|>2 cutoffs.

Gene ontology analysis was performed using the online functional classification tool Database for Annotation, Visualization and Integrated Discovery (DAVID; http://david.abcc.ncifcrf.gov/summary.jsp). Further analysis of the macrophage transcriptomes was performed in R v3.4.3 using Bioconductor v3.6. Zebrafish Ensembl gene IDs were converted to Entrez Gene IDs using the R package org.Dr.eg.db v3.5.0. The enriched pathways in different groups were determined by comparing the statistically differentially expressed genes against the KEGG zebrafish database using the kegga() function from the edgeR package v3.20.7. Finally, gene expression data were mapped into significantly enriched KEGG pathways using pathview v1.18.0.

### Statistical analysis

Statistical analysis was performed using GraphPad Prism 7 by one-way or two-way ANOVA (Figs 1, 2, 3, 7 and 8A, Fig. S4), Kolmogorov–Smirnov test (Fig. 8D,E) or two-tailed *t*-test (Fig. 4). Significance was accepted at *P*<0.05 and different significance levels are indicated: *P<0.05; **P<0.01; ***P<0.001; ****P<0.0001.

## Supplementary Material

Supplementary information
