# Supplementary material for: Glucocorticoids inhibit macrophage differentiation towards a pro-inflammatory phenotype upon wounding without affecting their migration
Source: Dis Model Mech. 2019 May 30;12(5):dmm037887. doi: 10.1242/dmm.037887 (PMC6550045; doi:10.1242/dmm.037887)
Supplement: Supplementary information [file dmm-12-037887-s1.pdf]

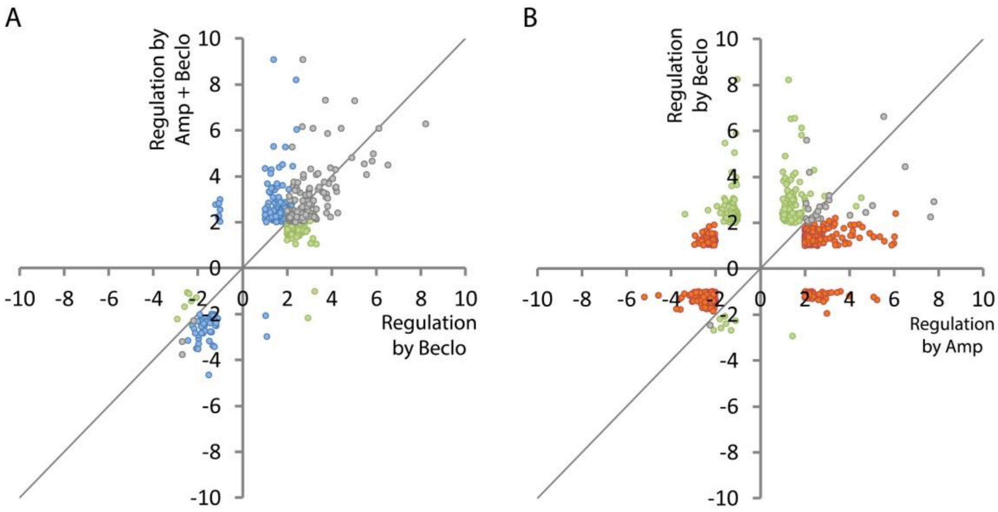

Figure S1. Scatter plot showing the difference and overlaps in gene regulation between different treatments. A. Comparison between beclomethasone and combined beclomethasone and amputation treatment. For all genes showing significant regulation upon beclomethasone (green and grey dots) or the combined beclomethasone and amputation treatment (blue and grey dots), the fold change due to beclomethasone and amputation treatment was plotted as a function of the fold change due to beclomethasone. The plot shows a large group of genes (grey dots) regulated by both treatments. B. Comparison between amputation and beclomethasone treatment. For all genes showing significant regulation upon amputation (red and grey dots) or beclomethasone treatment (green and grey dots), the fold change due to beclomethasone treatment was plotted as a function of the fold change due to amputation. The plot shows a very small group of genes (grey dots) regulated by both treatments. The grey line indicates the point at which the two different treatment have the same effect. Significantly regulated genes were selected by using a p.adj<0.05 and |FoldChange|>2 cutoff.

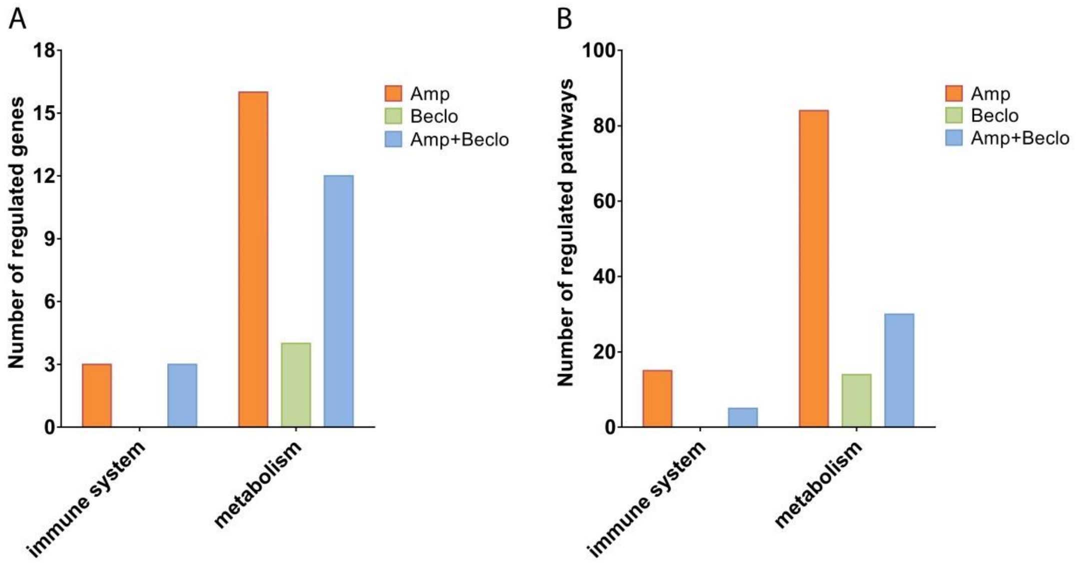

Figure S2. Gene ontology analysis of RNA sequencing experiment. A. The number of KEGG pathways overrepresented in clusters of genes significantly regulated by amputation, beclomethasone, and amputation+beclomethasone. Amputation regulated pathways involved in the immune system and in metabolism. Amputation+beclomethasone treatment regulated a lower number of regulated pathways involved in both the immune system and metabolism. B. The number of regulated genes involved in the overrepresented pathways. Amputation regulated genes involved in immune- and metabolism-related pathways. Amputation+beclomethasone regulated a lower number of genes involved in these pathways.

Disease Models & Mechanisms • Supplementary information

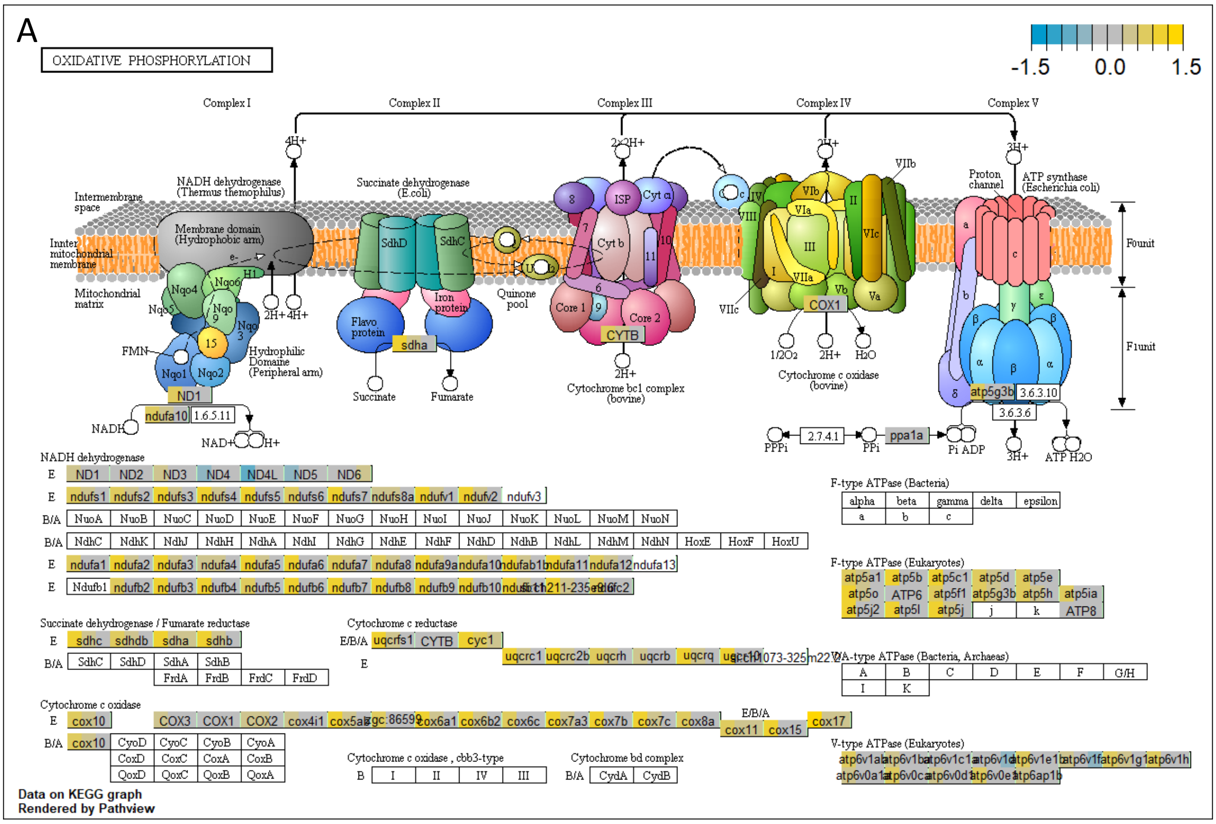

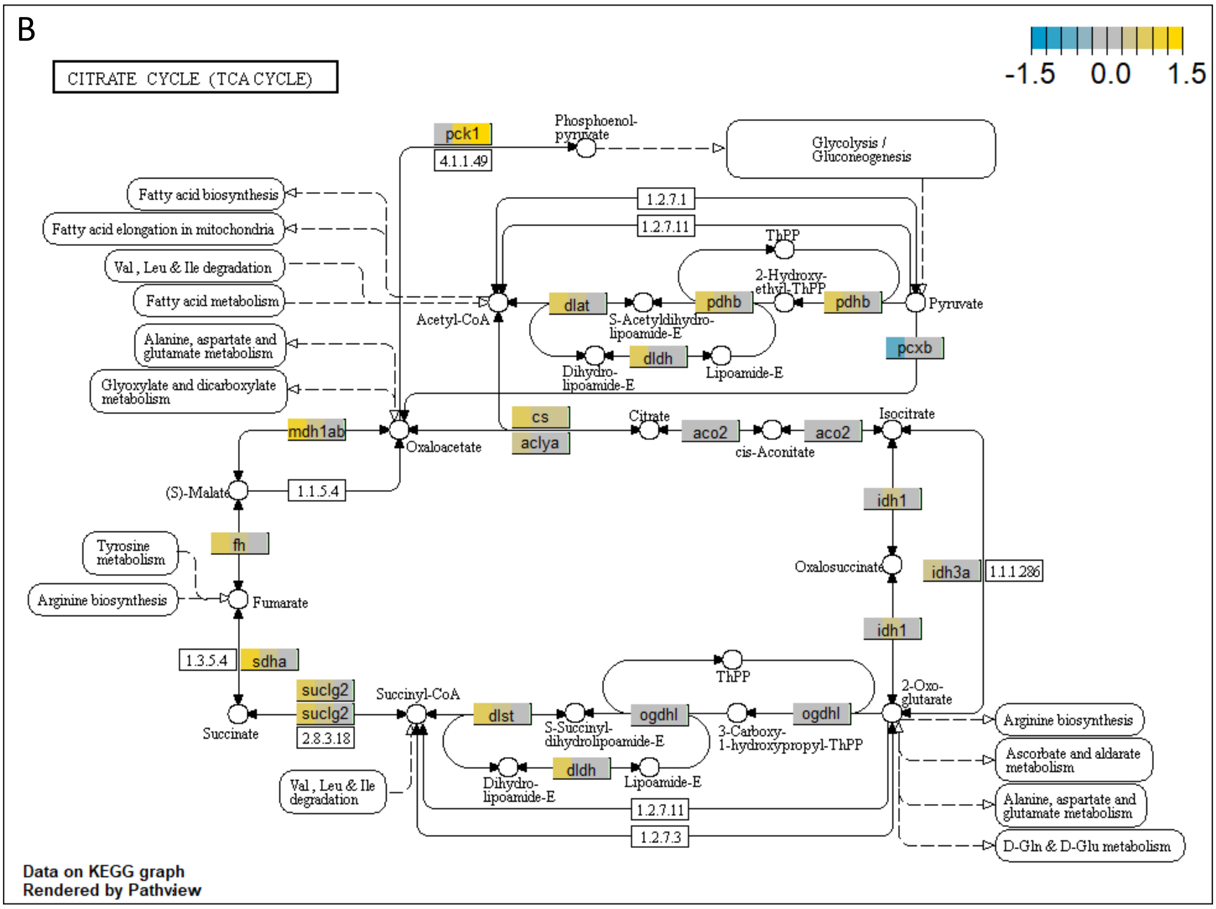

Disease Models & Mechanisms • Supplementary information

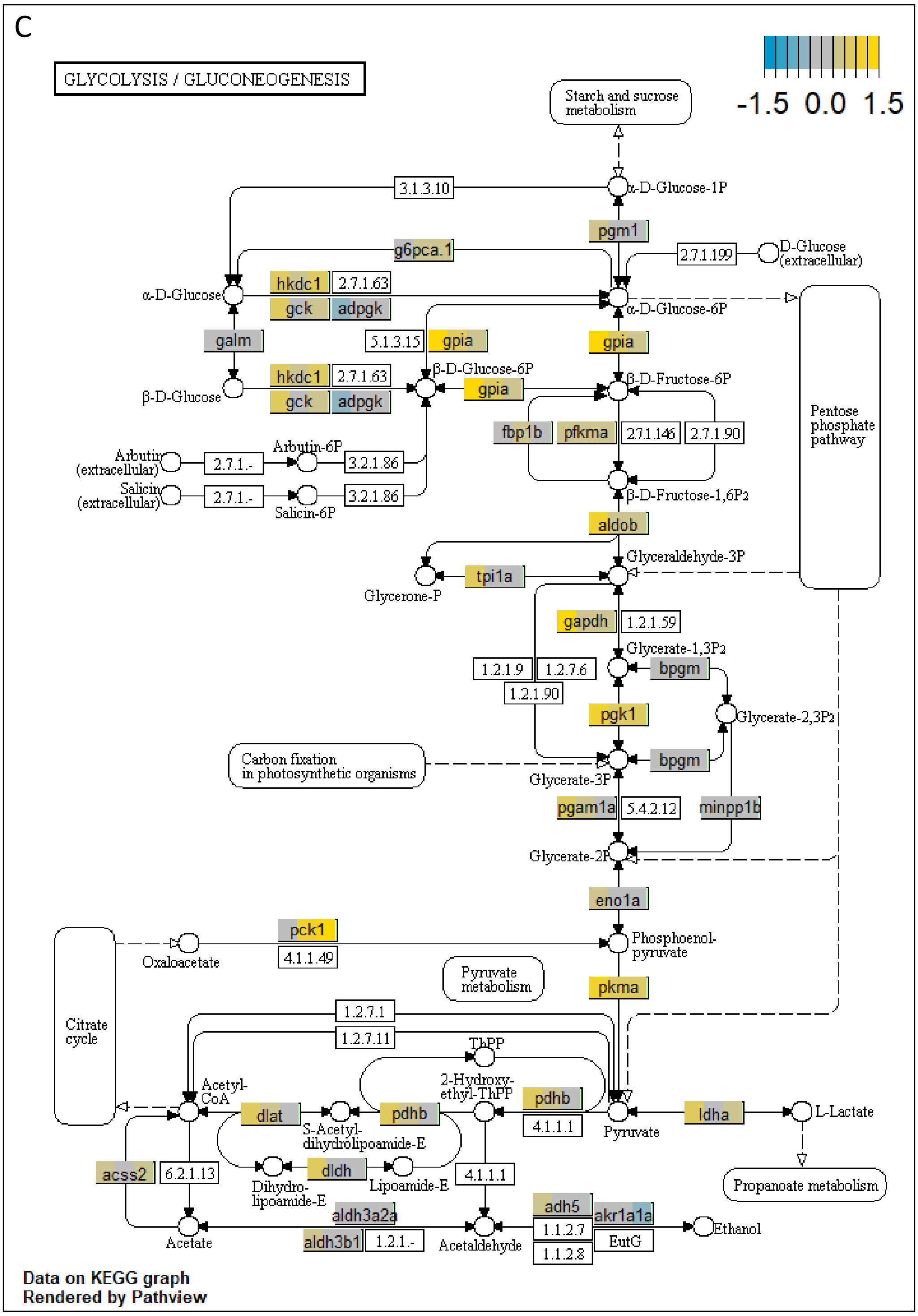

Figure S3. Significantly enriched KEGG pathways mapped with gene expression level. In the oxidative phosphorylation (A), Citrate cycle (TCA cycle) (B) and Glycolysis/ Gluconeogenesis (C) pathways, the fold change of the genes is indicated by different colour and the intensity of the colour as shown in the scale. The three colour shown in one gene indicated the logarithm fold change of amputation, beclomethasone and amputation+beclomethasone treatment respectively. The maps show that the vast majority of the amputation-induced changes in gene expression are inhibited by beclomethasone treatment in these pathways.

Disease Models & Mechanisms • Supplementary information

A

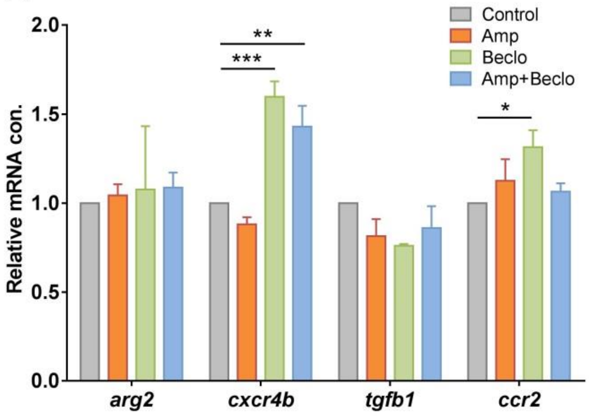

Figure S4. Expression levels of immune-related genes in FACS-sorted macrophages, determined by qPCR for *arg2, cxcr4b, tgfb1, ccr2* (A) at 24hpa in 3 dpf larvae. The expression level of *cxcr4b* was increased by beclomethasone treatment. Statistical analysis were performed by ANOVA with a Fisher's LSD post hoc. Values shown are the means ± s.e.m. of three independent experiments. Statistical significance is indicated by: * $P<0.05$; ** $P<0.01$; *** $P<0.001$.

Disease Models & Mechanisms • Supplementary information

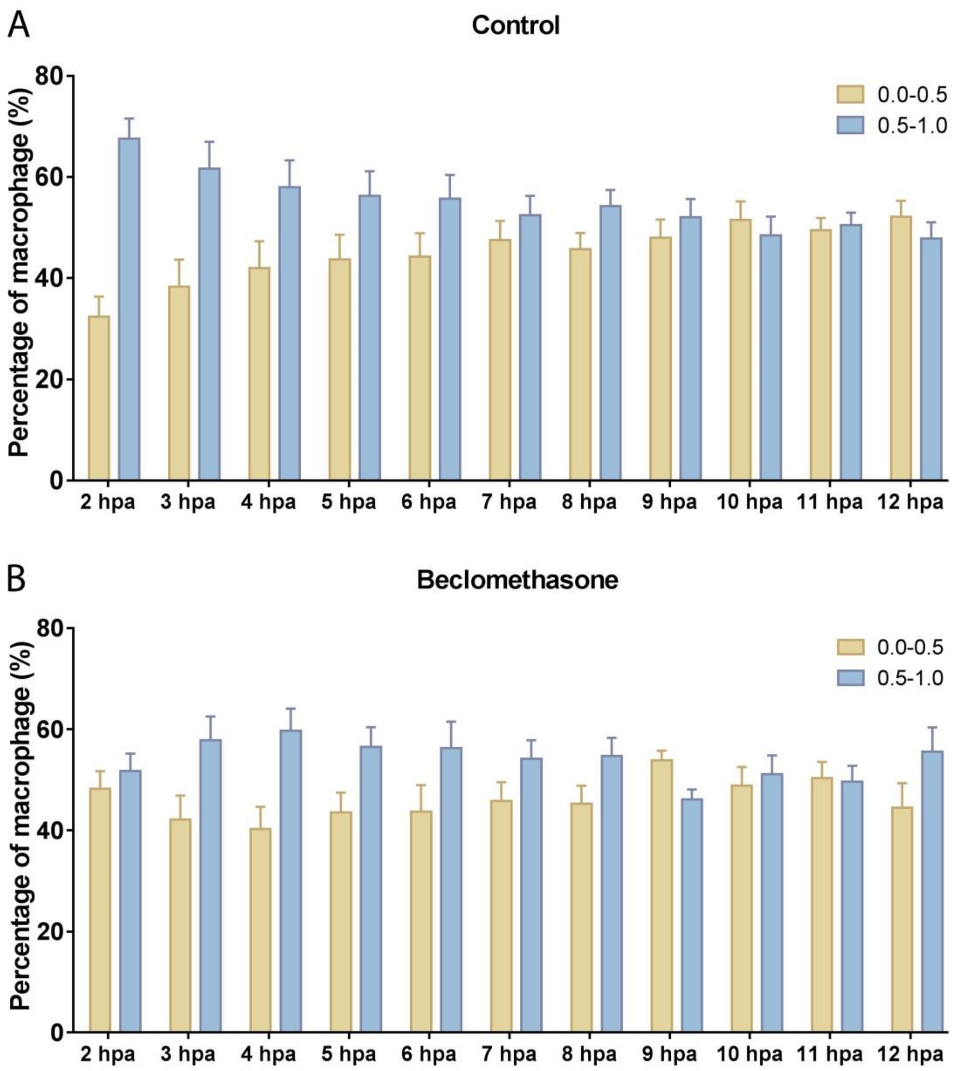

Figure S5. Effect of beclomethasone on the morphology of macrophages. The percentage of macrophages with different levels of circularity (0.0-0.5 and 0.5-1.0) were determined over time for the control group (A) and beclomethasone-treated group (B). In the control group, an increase in the percentage of macrophages with a circularity level between 0.0 and 0.5 and a decrease in the percentage of macrophages with a level between 0.5 and 1.0 was observed. In the beclomethasone-treated group, these changes were not observed. Values shown are means ± s.e.m..

Disease Models & Mechanisms • Supplementary information

Table S1. KEGG-pathways, based on RNA sequencing analysis.

Amputation

Term	Count	%	P-value	Fold Enrichment
**Immune system**				
NOD-like receptor signaling pathway	6	1.03	2.89E-02	3.43
Toll-like receptor signaling pathway	8	1.37	6.13E-02	2.26
Cytokine-cytokine receptor interaction	11	1.89	6.58E-02	1.88
Jak-STAT signaling pathway	8	1.37	9.53E-02	2.04
**Metabolism**				
Oxidative phosphorylation	28	4.81	2.08E-13	5.60
Carbon metabolism	23	3.95	2.72E-10	5.15
Biosynthesis of antibiotics	31	5.33	4.11E-10	3.73
Metabolic pathways	83	14.26	1.20E-09	1.84
Glycolysis / Gluconeogenesis	17	2.92	3.07E-09	6.52
Biosynthesis of amino acids	13	2.23	3.87E-05	4.28
Arginine and proline metabolism	10	1.72	1.33E-04	5.00
Pentose phosphate pathway	7	1.20	6.51E-04	6.32
Citrate cycle (TCA cycle)	6	1.03	5.67E-03	5.09
Fructose and mannose metabolism	6	1.03	1.42E-02	4.10
Pyruvate metabolism	6	1.03	1.73E-02	3.91
Galactose metabolism	5	0.86	2.82E-02	4.24
Insulin resistance	10	1.72	4.62E-02	2.11
Glycine, serine and threonine metabolism	5	0.86	7.43E-02	3.11
Propanoate metabolism	4	0.69	7.54E-02	4.00
RNA degradation	7	1.20	8.06E-02	2.31
**Others**				
Cardiac muscle contraction	13	2.23	1.30E-04	3.79

Disease Models & Mechanisms • Supplementary information

Amputation & Beclomethasone

Term	Count	%	P-value	Fold Enrichment
**Immune system**				
Toll-like receptor signaling pathway	6	1.97	8.62E-03	4.64
**Metabolism**				
Insulin signaling pathway	10	3.28	3.19E-04	4.45
Starch and sucrose metabolism	5	1.64	6.92E-04	11.96
Insulin resistance	8	2.62	1.49E-03	4.60
Steroid hormone biosynthesis	4	1.31	1.16E-02	8.27
FoxO signaling pathway	7	2.30	2.16E-02	3.15
Biosynthesis of antibiotics	8	2.62	2.94E-02	2.63
Arginine biosynthesis	3	0.98	3.76E-02	9.57
Glyoxylate and dicarboxylate metabolism	3	0.98	6.69E-02	6.96
Galactose metabolism	3	0.98	6.69E-02	6.96
Glycolysis / Gluconeogenesis	4	1.31	6.72E-02	4.19
Retinol metabolism	3	0.98	8.94E-02	5.89
Alanine, aspartate and glutamate metabolism	3	0.98	9.33E-02	5.74
**Others**				
Adipocytokine signaling pathway	5	1.64	2.43E-02	4.45

Beclomethasone

Term	Count	%	P-value	Fold Enrichment
**Metabolism**				
FoxO signaling pathway	8	4.08	1.48E-03	4.57
Insulin signaling pathway	8	4.08	1.59E-03	4.51
Insulin resistance	6	3.06	1.06E-02	4.38
Glyoxylate and dicarboxylate metabolism	3	1.53	4.35E-02	8.82
Steroid hormone biosynthesis	3	1.53	5.35E-02	7.87
**Others**				
Focal adhesion	7	3.57	3.80E-02	2.74
Adipocytokine signaling pathway	4	2.04	5.57E-02	4.51

Disease Models & Mechanisms • Supplementary information

Table S2. Sequences of Primers used in qPCR reactions.

Gene name	Gene accession	Sequence (5'-3')
ppail	ENSDARG00000103994	**Fw:** CATCCACAACCTTCCCGAACAC<br>**Rv:** ACACTGAAACACGGAGGCAAAG
ccl2	ENSDARG00000041835	**Fw:** GTCTGGTGCTCTTCGCTTTC<br>**Rv:** TGCAGAGAAGATGCGTCGTA
cxcl11aa	ENSDARG00000100662	**Fw:** ACTCAACATGGTGAAGCCAGTGCT<br>**Rv:** CTTCAGCGTGGCTATGACTTCCAT
il8	ENSDARG00000104795	**Fw:** TGTGTTATTGTTTTCCTGGCATTTC<br>**Rv:** GCGACAGCGTGGATCTACAG
cxcl18b	ENSDARG00000075045	**Fw:** TCTTCTGCTGCTGCTTGCGGT<br>**Rv:** GGTGTCCCTGCGAGCACGAT
il6	ENSDARG00000102318	**Fw:** CGCTAAGGCAACTGGAAGAC<br>**Rv:** CCAGACCACTGGGAAACACT
il1b	ENSDARG00000098700	**Fw:** TGTGTGTTTGGGAATCTCCA<br>**Rv:** CTGATAAACCAACCGGGACA
tnfa	ENSDARG00000009511	**Fw:** ACCAGGCCTTTTCTTCAGGT<br>**Rv:** TTTGCCTCCGTAGGATTCAG
mmp9	ENSDARG00000042816	**Fw:** CATTAAAGATGCCCTGATGTATCCC<br>**Rv:** AGTGGTGGTCCGTGGTTGAG
arg2	ENSDARG00000039269	**Fw:** AAGGCCATTCTCAGCAGTGT<br>**Rv:** AGGTTTCCCGAAGGTGAAGT
cxcr4b	ENSDART00000061499	**Fw:** GCGACCTCTCAGTCAGCAAT<br>**Rv:** TCACAAGCACCACAAGTCCA
tgfb1	ENSDARG00000041502	**Fw:** CAACCGCTGGCTCTCATTTGA<br>**Rv:** ACAGTCGCAGTATAACCTCAGCT
ccr2	ENSDARG00000079829	**Fw:** TGGCAACGCAAAGGCTTTCAGTGA<br>**Rv:** AGGTTTCCCGAAGGTGAAGT

Disease Models & Mechanisms • Supplementary information

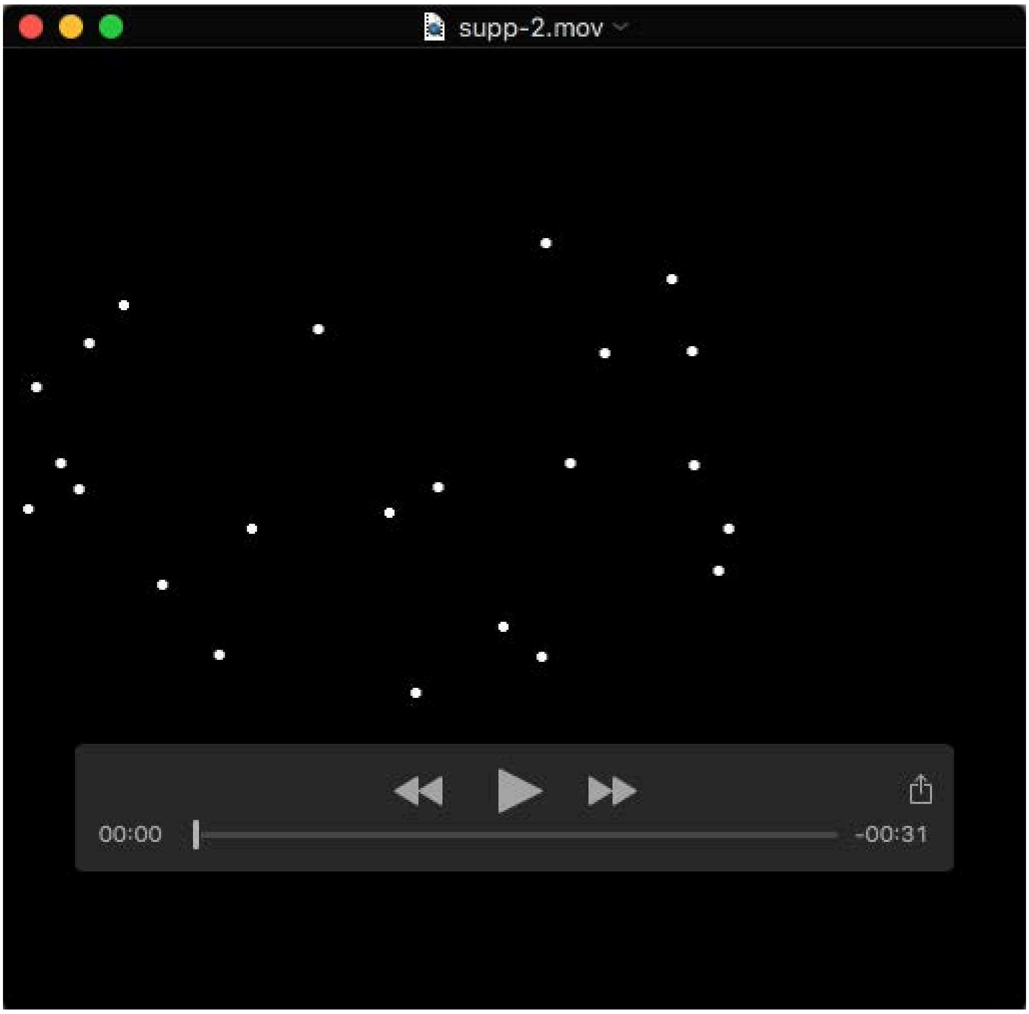

Movie 1. Macrophage migration upon tail amputation. Tracks of macrophages migrating between 1.5 and 5.5 hpa in 3 dpf larvae in the vehicle-treated group. Confocal microscopy images were analyzed using ImageJ with custom-made plugins, developed by Dr. Joost Willemse (Leiden University), for localization and tracking of cells.

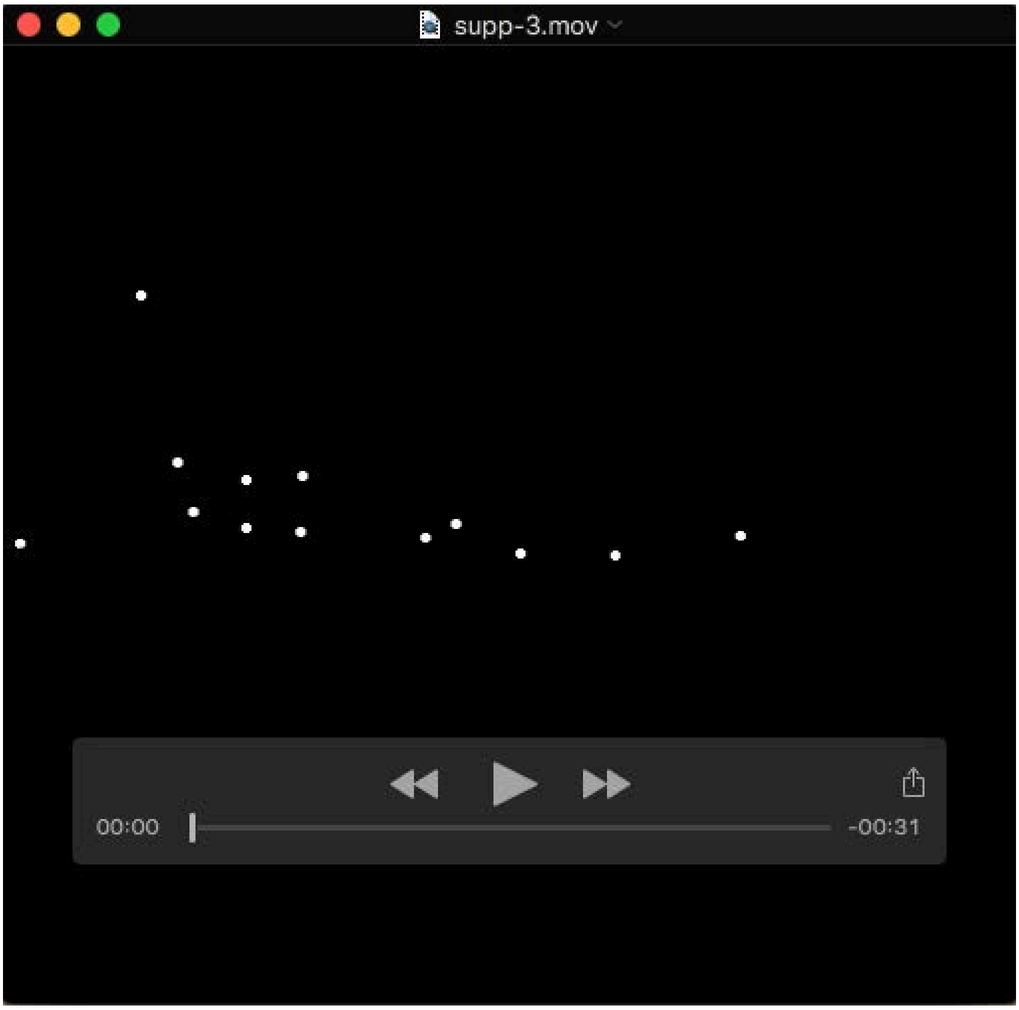

Movie 2. Neutrophil migration upon tail amputation. Tracks of neutrophils migratimg between 1.5 and 5.5 hpa in 3 dpf larvae in the vehicle-treated group. Confocal microscopy images were analyzed using ImageJ with custom-made plugins, developed by Dr. Joost Willemse (Leiden University), for localization and tracking of cells.

Disease Models & Mechanisms • Supplementary information
